# Simple yet effective methods to probe hydrogel stiffness for mechanobiology

**DOI:** 10.1038/s41598-021-01036-5

**Published:** 2021-11-22

**Authors:** Alessandro Gandin, Yaswanth Murugesan, Veronica Torresan, Lorenzo Ulliana, Anna Citron, Paolo Contessotto, Giusy Battilana, Tito Panciera, Maurizio Ventre, A. Paolo Netti, Lucia Nicola, Stefano Piccolo, Giovanna Brusatin

**Affiliations:** 1 Department of Industrial Engineering, University of Padova and INSTM, via Marzolo 9, 35131 Padova, Italy; 2 Department of Industrial Engineering, University of Padova, via Marzolo 9, 35131 Padova, Italy; 3 Department of Molecular Medicine, University of Padova, via Ugo Bassi 58/B, 35131 Padova, Italy; 4 Department of Chemical, Materials and Industrial Production Engineering, University of Naples Federico II, 80125 Naples, Italy; 5 Center for Advanced Biomaterials for Healthcare@CRIB, Istituto Italiano Di Tecnologia, L.go Barsanti e Matteucci 53, 80125 Naples, Italy

**Keywords:** Biomaterials - cells, Gels and hydrogels, Characterization and analytical techniques

## Abstract

In spite of tremendous advances made in the comprehension of mechanotransduction, implementation of mechanobiology assays remains challenging for the broad community of cell biologists. Hydrogel substrates with tunable stiffness are essential tool in mechanobiology, allowing to investigate the effects of mechanical signals on cell behavior. A bottleneck that slows down the popularization of hydrogel formulations for mechanobiology is the assessment of their stiffness, typically requiring expensive and sophisticated methodologies in the domain of material science. Here we overcome such barriers offering the reader protocols to set-up and interpret two straightforward, low cost and high-throughput tools to measure hydrogel stiffness: static macroindentation and micropipette aspiration. We advanced on how to build up these tools and on the underlying theoretical modeling. Specifically, we validated our tools by comparing them with leading techniques used for measuring hydrogel stiffness (atomic force microscopy, uniaxial compression and rheometric analysis) with consistent results on PAA hydrogels or their modification. In so doing, we also took advantage of YAP/TAZ nuclear localization as biologically validated and sensitive readers of mechanosensing, all in all presenting a suite of biologically and theoretically proven protocols to be implemented in most biological laboratories to approach mechanobiology.

## Introduction

Hydrogels play a major role in mechanobiology allowing investigations on how cells sense substrate rigidity and tune their behavior accordingly. A wealth of studies used hydrogels to demonstrate the overarching impact that physical cues exert on almost every aspect of cell behavior, from the control of cell proliferation, survival, stemness and differentiation^1,2^. To mimic the mechanical properties of natural soft tissues, the elastic modulus of these substrates should be in the range < 1 kPa to few 10 kPa. In this range, rigidity determination is challenging, as hydrogels are special materials in the realm of soft matter physics and material science: they are much softer than common materials (ceramics, metals and plastics) and of classic rubbers, displaying a non-linear behavior under most strain conditions^3,4^. Moreover, as hydrogels easily break, slip and deform under their own weight, measuring their stiffness is challenging using typical traction or bending assays. Appreciation of even slight differences in elastic modulus is also desired for meaningful experiments of mechanobiology; for example, changes in ECM stiffness of fractions of kPa has been recently shown to have profound consequences in cell biology, discriminating between oncogene-induced transformation and normal behavior of epithelial cells^5^. This further raises the bar for the need of precise, sensitive, reproducible and yet straightforwardly applicable measurements of hydrogel rigidity. The latter is generally measured by methods such as rheological measurements or atomic force microscopy (AFM) that are highly reliable to measure slight variation of stiffnesses, for both low (< 1 kPa) and high (> tens of kPa) stiffnesses^6–9^. However, simple, cheap and high throughput techniques would be desirable for a systematic testing of hydrogel substrate rigidities. Here we propose the use of static macroindentation and micropipette aspiration as means to fill this gap. We offer detailed protocols for implementing these two simple techniques, enabling a routinely measurement of hydrogel stiffness for mechanobiology applications also by cell biology teams that are new to fabrication and material science. Here we show their effectiveness over the physiological range of natural tissues and validate them through a comparison with traditional, yet manyfold more expensive and complex methodologies, such as AFM, rheometry and compression, with consistent results.

## Results

**Figure 1:**
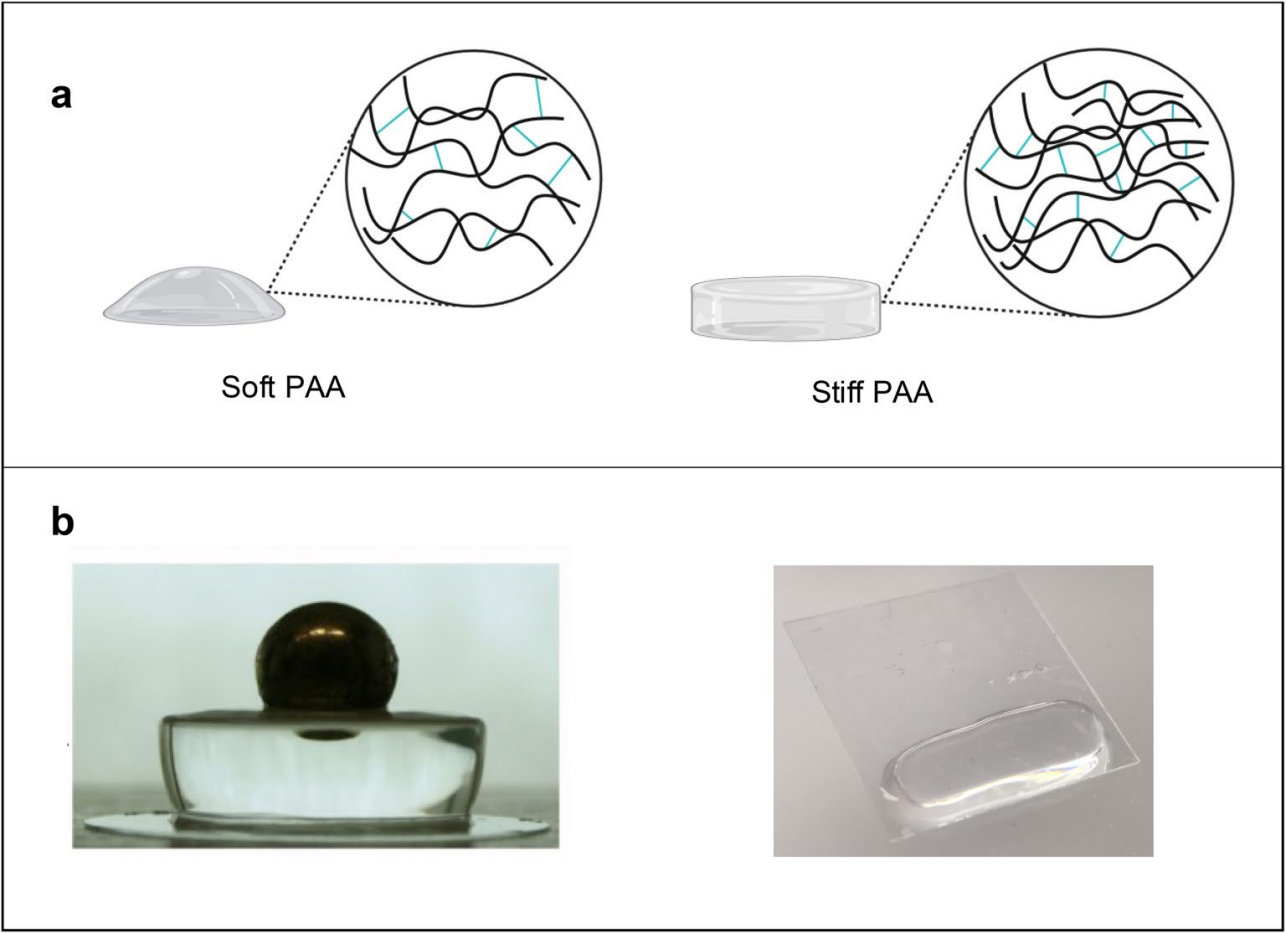
(**a**) Scheme of PAA hydrogels soft and stiff and (**b**) example of sample geometries used for macroindentation (left) and micropipette aspiration (right) tests.

We focused on the development of two simple methods to measure the elastic modulus of hydrogels: static macrosphere indentation and micropipette aspiration. With these tools, hydrogels are deformed under the application of a pressure, which derives from the sphere weight or the aspiration pressure, and the deformation of the gel is quantified and related to the elastic modulus of the gel as described below. Protocols for their experimental realization, set-up details and all technical components are supplied in the methods. In this study we have applied these tools on classic Polyacrylamide (PAA) hydrogels, synthesized with variable rigidities, and the resulting values of stiffnesses have been compared with those obtained using more sophisticated techniques. Six PAA hydrogels formulations named PAA1-6 were prepared as previously reported^10,11^, with increasing crosslinking densities (see Fig. 1a for the scheme of a soft and a stiff hydrogel structure, and Table 1 for Acrylamide (AA) and Bis-acrylamide (BA) copolymerization), in order to cover a range of stiffnesses from < 0.7 to 40 kPa (as measured by AFM in^11^). To prepare gels for macroindentation (Fig. 1b left), the precursor solutions were polymerized in a 4 mm height 48-well polystyrene plate (diameter 10 mm). For micropipette aspiration (Fig. 1b right), the precursor solution was pipetted inside PDMS rings (thickness of 1–2 mm, area of about 1cm^2^) placed between a Kapton film (non-PAA adhesive) and a functionalized (PAA adhesive) coverslip (see “Methods” ). Once polymerized, all gels were left overnight to reach the swelling equilibrium before performing the mechanical tests.

**Table 1:** Experimental data of macroindentation tests on the 6 PAA hydrogels. D is measured for one indentation picture. E^NH-fhd^ is calculated for a minimum of 10 measurements made on the same gel and ΔE represents the standard deviation.

Hydrogel	*A/BA%wt	Indenter	*δ/h* min	*δ/h* max	E^NH-fdh^ (kPa)	ΔE^NH-fdh^ (kPa)
PAA1	4/0.03	**SP**	0.195	0.227	0.14	0.01
PAA2	4/0.06	**SS**	0.240	0.274	1.30	0.09
PAA3	4/0.1	**SS**	0.157	0.194	2.69	0.24
PAA4	5/0.15	**SS**	0.073	0.089	9.58	0.88
PAA5	5/0.225	**SI**	0.314	0.364	11.86	0.97
PAA6	8/0.48	**SI**	0.115	0.150	56.37	8.15

*In this column the wt% of the two monomers are reported. A represent the monofunctional acrylates Acrylamide and BA the bifunctional Bis-acrylamide. For PAA-OH gels, A represent the sum of the monofunctional acrylates Acrylamide and N-hydroxyethyl acrylamide (HEA). The amount of HEA is fixed at 1 mM. Sphere radius (R) and indentation force (F) are reported in Table SI.

### Static macrosphere indentation

Multiple efforts over the last two decades have been dedicated to developing accessible ways of measuring gel mechanics, including methods based on material indentation. The latter can be applied at different scales, from macro to microindentation, in static or dynamic strain conditions^9,12–16^. Original work from the Wang laboratory carried out elasticity measurements by hanging weights from PAA gels and measuring gel deformation. That said, a drawback of microindentation is the need of complex imaging procedures^10,17–20^. To overcome this limitation, here we have applied a macroindentation procedure that can be easily applied using simple equipment. In principle, static macroindentation, measuring the penetration of the gel by a sphere with diameter of the order of millimeters, can be easily performed using a common digital camera and a microscope. In practice, however, this requires the availability of suitable mathematical tools to obtain the Young modulus (E) from imaging data, that are not currently available. Indeed, an experimentally validated theoretical model is essential for a correct determination of E, considering that indentation causes a complex stress state inside the sample. Unfortunately, few studies have addressed this problem on hydrogels and, when done, refer to a limited range of stiffnesses and sample geometries^15,16^.

**Figure 2:**
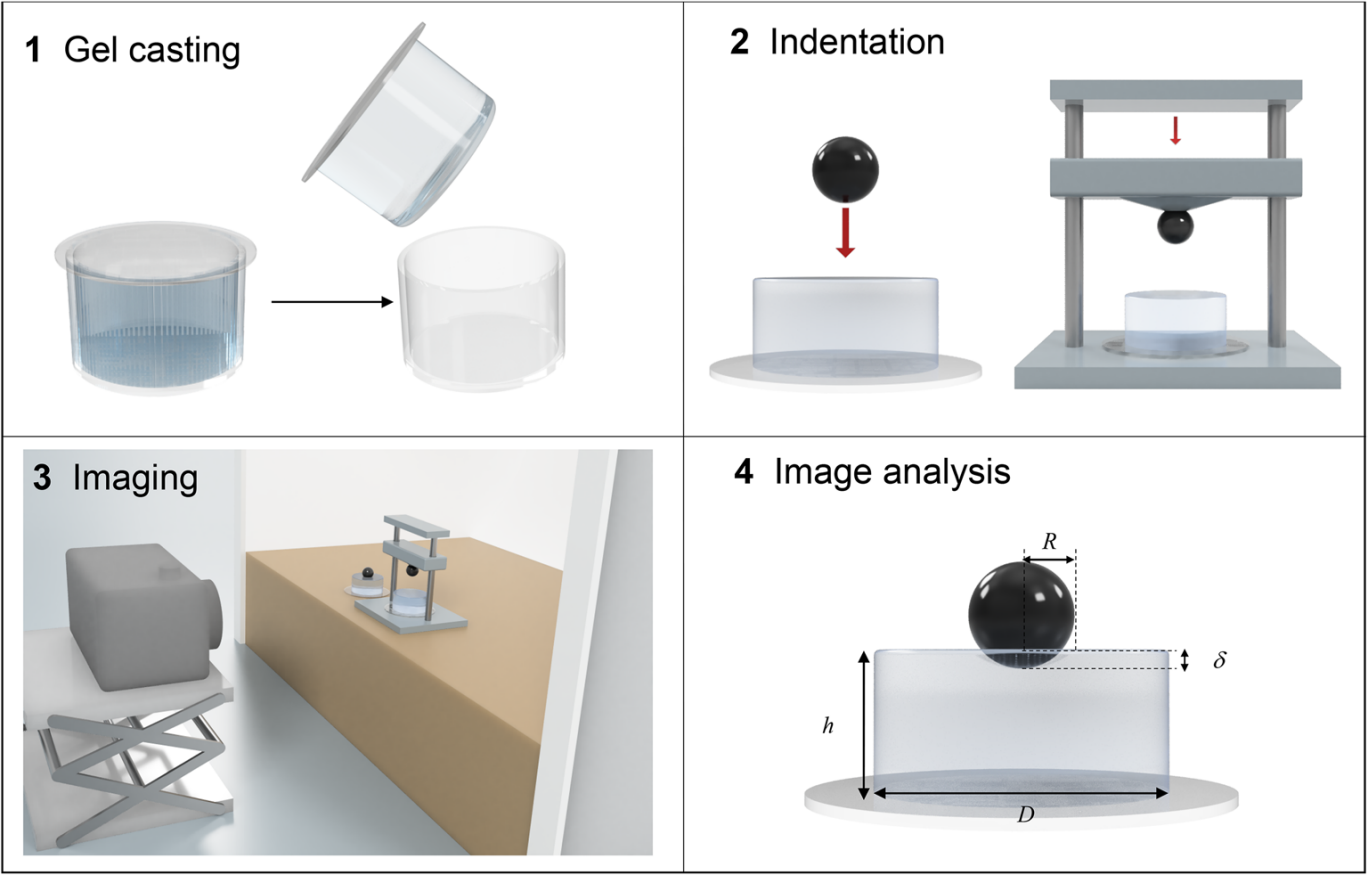
Workflow of the macroindentation process. 1. Preparation of the gel with a cylindrical shape 2. The indenting sphere is put on the hydrogel surface eventually overloaded as shown in the third step 3. The image is captured with a camera 4. And analyzed to determine *δ, h, D* with a MATLAB program.

To advance in this direction, we carried out macroindentations and implemented the theoretical models underlying interpretation of macroindentation data, including the finite geometrical size effects of the experimental samples, all in all allowing to perform straightforward static indentation at the macro-scale and readily derive the E value of gels. Figure 2 shows a workflow of the static macroindentation process. The hydrogels were prepared in multiwell plate as described above, resulting in cylindrical shaped gels of height *h* and diameter *D*, and indented using a rigid sphere of radius *R* placed on top. The image is captured immediately so avoiding drying of the hydrogel. Figure S1 shows the set-up of the static macroindentation process and Fig. S2 an example of pictures for each of the 6 PAA hydrogels, from which the image analysis is made.

The experimental data (indentation depth *δ*, gel height *h* and gel diameter *D*) are measured from the picture taken using any common digital camera and repeating the measure 10 times. Images were analysed with the MATLAB software, to obtain *δ*, *h* and *D*. From each set of data, the calculation of the elastic modulus of the hydrogel is made using Eq. (1). The Young’s modulus is given as a function of the experimental data: the indentation force *F* (i.e., the weight of the sphere) and the sphere radius *R* (details on the derivation of Eq. (1) are given below).

Table 1 reports, for the 6 PAA gels, the achieved maximum and minimum values of *δ/h* ratio and the mean value of the 10 elastic moduli calculated for each gel, indicated as E^NH-fdh^, using Eq. (1). As indicated in Table 1, a different indenter is used for each gel. In fact, we proceeded with macroindentation tests on each PAA hydrogels using different spheres, i.e. spheres of different weight and size as reported in Table S1, and obtaining different values of elastic modulus, as reported in Table S2. The most appropriate indenter for each PAA gel was empirically selected from Table S2 to minimize reading errors.

1 $$E^{{NH - fdh}} = \frac{{9F}}{16R^{1/2}\delta^{3/2}} \times \frac{{f\left( {\frac{R}{h},\omega } \right)}}{{g\left( {\frac{\delta }{h},\frac{{Rh}}{{D^{2} }}} \right)}}$$

In Eq. (1), *f* and *g* represent correction factors to the Hertz contact theory, the easiest approach to evaluate the elastic modulus of the specimens from indentation data, which provides a simpler relationship between load *F*, indentation depth δ and radius of the sphere *R* (Eq. 2) but valid for a semi-infinite sample:

2 $$E^{H} = \frac{9F}{{16R^{1/2}\delta^{3/2}}}$$

The analytical expressions of correction factors f and g are given in Eqs. (3) and (4) respectively, and their relevance is discussed in the next section

3 $$f\left(R/h, \omega\right)= \frac {1+ 2.3\omega} {1+ 1.15\omega^{1/3}+\alpha\left(R/h\right) \omega + \beta \left(R/h\right)\omega^2}$$

3.1 $$\omega = \left( {R \delta}/{h^2}\right)^{3/2}$$

3.2 $$\alpha \left( {{\raise0.7ex\hbox{$R$} \!\mathord{\left/ {\vphantom {R h}}\right.\kern-\nulldelimiterspace} \!\lower0.7ex\hbox{$h$}}} \right) = 10.05 - 0.63\sqrt {{\raise0.7ex\hbox{$R$} \!\mathord{\left/ {\vphantom {R h}}\right.\kern-\nulldelimiterspace} \!\lower0.7ex\hbox{$h$}}} \times \left( {3.1 + {\raise0.7ex\hbox{${h^{2} }$} \!\mathord{\left/ {\vphantom {{h^{2} } {R^{2} }}}\right.\kern-\nulldelimiterspace} \!\lower0.7ex\hbox{${R^{2} }$}}} \right)$$

3.3 $$\beta \left( {{\raise0.7ex\hbox{$R$} \!\mathord{\left/ {\vphantom {R h}}\right.\kern-\nulldelimiterspace} \!\lower0.7ex\hbox{$h$}}} \right) = 4.8 - 4.23\left( {{\raise0.7ex\hbox{${h^{2} }$} \!\mathord{\left/ {\vphantom {{h^{2} } {R^{2} }}}\right.\kern-\nulldelimiterspace} \!\lower0.7ex\hbox{${R^{2} }$}}} \right)$$

4 $$g \left( {\frac{\delta }{h},\frac{Rh}{{D^{2} }}} \right) = 1 + \left( {\frac{Rh}{{D^{2} }}} \right)^{2} \left( { - 0.07 - 0.22\frac{\delta }{h}} \right) + \left( {0.14 - 0.33\frac{\delta }{h}} \right)\left( {\frac{\delta }{h}} \right) + \frac{Rh}{{D^{2} }}\left( {0.007 + \frac{\delta }{h}} \right)\left( { - 1.025 + 1.42\frac{\delta }{h}} \right)$$

The results show that, through this simple procedure and application of Eq. (1)—i.e. something readily applicable by any cell biology laboratory—we could measure a range of Young moduli from fractions of kPa to > 50 kPa, as such covering most of the physiological rigidity values of natural tissues. As shown below in Fig. 7, these values are in line with those obtained with other and more complex methods.

### Correction factors to the Hertz theory

In this paragraph we detail how Eq. (1), used to calculate the elastic modulus of hydrogels, is achieved. One must notice that Hertz theory on how to calculate rigidities from indentation data is based on some assumptions: one is that the specimen is assumed to be a linear-elastic incompressible half-space. Indeed, it can be safely assumed that the hydrogel is an incompressible material, as macroindentation experiments are typically carried out in a much shorter timescale than diffusion of the small solvent molecules in and out the gels network, and thus characterized by a Poisson’s ratio *ν* = 0*.*5. Another assumption of the Hertz theory is that the deformation is within the limits of small-slope and small-strain conditions is however unlikely satisfied in a macroindentation experiment where the specimen has finite and rather small dimensions with respect to the indenter. Indeed, the small slope conditions (defined by the relation *a*^2^ = *R δ*), are satisfied only for specific geometries of the contact problem. This is shown in Fig. S3, indicating for three different specimen dimensions expressed by *D/h* (namely *D/h* = 5, 2, and 1), the values of *R/D* and *δ* /D below which the small slope conditions are fulfilled. However, since our experimental data had *D/h* = 2 or higher (Fig. S3) they violate the small-slope assumption. Additionally, the measured strain *δ/h* in our experiments was always greater than 0.07 (refer to Table 1 for the experimental values). This indicates that the assumption of small strain, for which a linear dependence between stress and strain should be observed, is also not satisfied as also shown by uniform compression tests performed on our specimen (Fig. S4).

**Figure 3:**
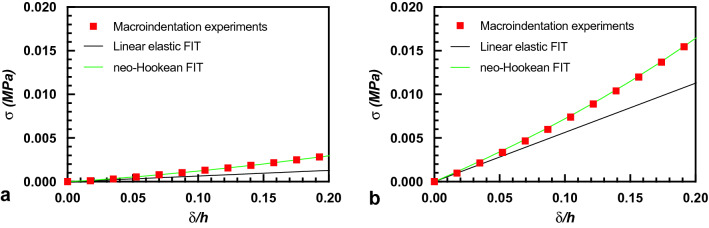
(**a**,**b**) Stress–strain curves obtained through uniform compression for soft PAA2 (**a**) and stiff PAA6 (**b**) hydrogels. The experimental results are better fitted by a Neo-Hookean constitutive behavior than by linear elasticity.

Thus, the Hertz equation cannot be used as it is in macroindentation, and a new model is necessary to calculate the elastic modulus of our specimens. To this end, it is important to first identify the material behavior that best describes the deformation response. While the literature often reports a linear elastic behavior for the hydrogels at low loading, non-linear elastic behaviour has also been reported and described with different models, such as neo-Hookean, Mooney-Rivlin, etc.^21,22^. To ascertain which of these non-linear models better resembles the hydrogel specimen response, we used the uniform compression tests of Fig. S4 and performed a fit of the data using different non-linear models. Two examples of the stress–strain response are reported in Fig. 3a,b together with the linear-elastic and the neo-Hookean fits to the data. From this figure, one can see that the non-linear neo-Hookean model well describes the material behaviour.

Therefore, we adopted the expression for the indentation proposed in the work by Long et al*.*^15,23^, in which hydrogels were, in fact, treated as neo-Hookean material. This expression, indicated as *E*^*NH-fh*^, accounts also for the finite thickness of the specimens, deformed by a spherical indenter:

5 $$E^{NH - fh} = \frac{9F}{{16R^{1/2}\delta^{3/2}}} \times f\left( {\frac{R}{h}, \omega } \right)$$

Notice that the equation reads like the Hertzian equation albeit for a correcting term, *f(R/h,w)* defined in Eq. (3) and valid for a slip condition, without adhesion between the gel and the indenter.

**Figure 4:**
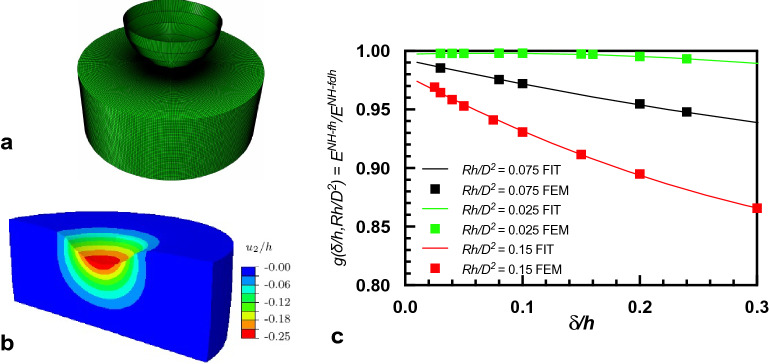
(**a**,**b**) FEM analysis was performed using ABAQUS choosing a finite element quad-mesh composed by 64,000 elements (**a**) Normal displacement shown for PAA6 (**b**). The simulation was performed to calculate both the elastic modulus and the normal displacement exerted on the hydrogel by the indenting sphere. (**c**) The evolution of correction factor *g*(*δ/h,Rh/D*^2^) with strain (*δ/h*) for different values of the finite diameter and also given by the empirical fit.

We then asked ourselves if the elastic response of our hydrogels is also affected by their lateral dimension, because hydrogel specimens do not only have a non-linear elastic behaviour and a finite height but also have a finite diameter, not considered in the Eq. (5). In this work, the effect of the finite diameter was assessed by means of Finite Element simulations performed using the commercial software ABAQUS (Fig. 4a-b). In the simulations, the indenter is modelled as a rigid sphere and the substrate is modelled as an incompressible neo-Hookean cylindrical solid, with the experimentally measured dimensions. Then, among the experimental data, the more severe values of indentation depths were chosen for each gel to simulate the indentation and derive the elastic modulus. In Fig. 4a,b, the finite element mesh is shown along with the normal displacement field of the deformed hydrogel. Data used for the simulation are reported in Table S3. Through this finite element simulations for different geometries and indentation depth, we can identify the conditions for which a correction is necessary and provide the correction in the form of a polynomial equation. The need of a correction factor for the diameter is found to depend on the *δ/h* ratio and the geometric factor *Rh/D*^2^, the dimensionless strain and dimensionless diameter respectively, as shown in Fig. 4c. The correction factor *g*(*δ/h,Rh/D*^2^) previously reported in Eq. (4) is so obtained, through the method of least square regression.

In essence, our Eq. (1) represents a modification of Long et al.^15^, that accounts for a finite height neo-Hookean material, augmented by a function of the two dimensionless arguments *δ/h* and *Rh/D*^2^*, g*(*δ/h,Rh/D*^2^).

To verify the extent of correction introduced by our model, that considers both finite diameter and non-elastic behavior, the elastic moduli obtained by our FEM simulations E^NH-fdh^ were compared with those calculated through different models, using the experimental data of Table S3. In particular, the calculation of the elastic modulus achieved by the Hertz model (E^H^, Eq. (2)), the model proposed by Long et al.^15^ (E^NH-fh^, Eq. (5)) and a model proposed by Dimitriadis^24^, E^H-fh^, that takes into account the finite height of the gel but in a linear elastic material (instead of neo-Hookean), were made and compared in Table 2. From the table it can be seen that the finite height correction is the most relevant one, about 20–30%, as it results from the comparison between the Hertz linear model and the other three calculated values, E^NH-fh^, E^H-fh^ and E^NH-fdh^, while the correction for a non-linear behavior, i.e., E^NH-fh^ and E^NH-fdh^, partially counteract the finite height correction. Besides, comparison of E^NH-fdh^ (our results) and E^NH-fh^ (calculated without taking into account a finite diameter) indicates that a correction lower than 7% is needed in our experimental conditions. In fact, in the experiments we conducted here, we used a sufficiently small indentation depth and large diameter (*Rh/D*^2^ = 0.05) that the results are not so far from Long’s equation. This would not be the case for experiments on smaller specimens or with a larger indentation depth, as shown by curves for *Rh/D*^2^ = 0.075 and *Rh/D*^2^ = 0.15 in Fig. 4c. In that case the final values would be more affected by the correction function *g*(*δ/h,Rh/D*^2^).

**Table 2:** Elastic modulus estimated from the classical Hertz theory *E*^H^, Hertz theory corrected for finite height *E*^H-fh^, Hertz theory corrected for neo-Hookean non-linearity and finite height *E*^NH-fh^, Hertz theory corrected for neo-Hookean nonlinearity and finite dimensions (finite height and finite diameter) *E*^NH-fdh^.

Sample	*E*^*H*^ (kPa)	$E^{H - fh}$ (kPa)	$E^{NH - fh}$ (kPa)	$E^{NH - fdh}$ (kPa)	$\frac{{E^{NH - fdh} - E^{NH - fh} }}{{E^{NH - fdh} }}$
PAA1	0.18	0.12	0.13	0.14	7%
PAA2	1.82	1.05	1.15	1.20	4%
PAA4	3.46	2.14	2.27	2.27	0%
PAA5	11.39	8.29	8.55	8.92	4%
PAA6	11.39	9.11	10.36	11.23	7%
PAA7	67.91	44.91	47.11	50.43	7%

### Micropipette aspiration

Micropipette aspiration^25^ is also a simple and cheap technique. The main advantage of this method is its versatility, as it can be used to measure stiffness of cells and soft natural tissues^22,23,26^, ex vivo or in vivo^27,28^. In literature, few examples of its use on siloxane polymers or stiff gels are reported^29,30^, but theoretical models have been exhaustively described to correlate tissue deformation, aspiration pressures and geometrical parameters, and can be extended to soft hydrogels. Here we advance by providing set-up protocols to build micropipette aspiration, so far poorly described in literature, and offer technical details and instruction to perform reliable measurements.

**Figure 5:**
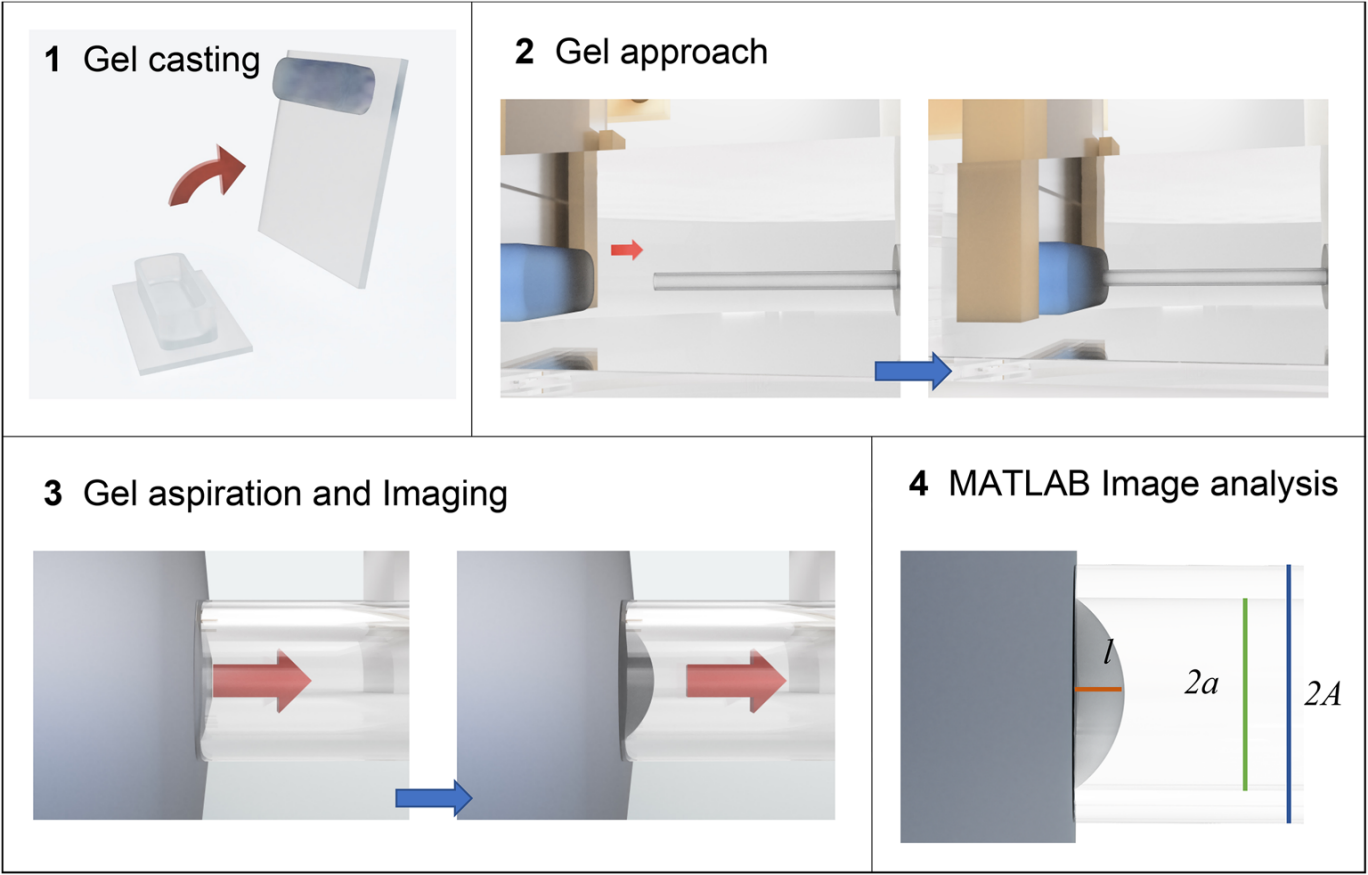
Workflow of the macroindentation process. 1. Preparation of the gel inside a PDMS ring 2. The gel approaches the micropipette cross section until contact is reached and pressure is applied 3. The image is captured with an inverted microscope 4. and analyzed to determine the internal radius *a * and the aspiration length *l* with a MATLAB program.

Figures 5 and S5 show a workflow and a picture of the micropipette aspiration apparatus respectively. To perform these analyses, we designed and fabricated a device consisting of a testing head that holds a capillary (external radius *A* = 0.5 mm and internal radius *a* = 0.375 mm) and moves the sample in the xy plane, a circuit enclosure containing the motors controller and the pressure sensors and a syringe pump to exert a controlled pressure on the sample (see methods for a detailed description of the system).

The mechanical characterization is made mounting the sample with the free surface perpendicular to the glass capillary and moving the sample until a full contact between the surface and the capillary section is achieved. Then, a given negative pressure is applied, through an aspiration pipette, that induces a small convexity in the gel surface, from which a certain aspiration length *l* can be observed. As a certain pressure *P* is reached, a picture is captured by a microscope imaging software, and the aspirated length l can be measured using an image analysis software. Figure S6 shows example of pictures for each of the 6 PAA hydrogels, from which the image analysis is made.

The local deformation of the hydrogel in the pipette is then related to the applied pressure *p* and the internal radius of the pipette *a*, using an explicit expression developed through FEM to calculate the hydrogel elastic modulus, as reported by Zhang et al.^30^. In this model, friction between gel and the internal surface of the capillary should be avoided. For this, in our experiments, a fluorinated functionalization was made in the internal surface of the pipette.

Moreover, the model discriminates between linear or nonlinear elastic deformations. The proposed parameter to discriminate the two behaviors is *l/a*, set at values *l/a* < 0.3 or *l/a* > 0.3 for the linear or non-linear regimes respectively, and the relative equations used to calculate E, named E^PA^, are the following:

6 $$E^{PA} = \frac{p}{1.07{\frac{l}{a}}}$$

7 $$E^{PA} = \frac{p}{{0.872{\frac{l}{a}} + 0.748\left( {\frac{l}{a}} \right)^{2} }}$$

**Figure 6:**
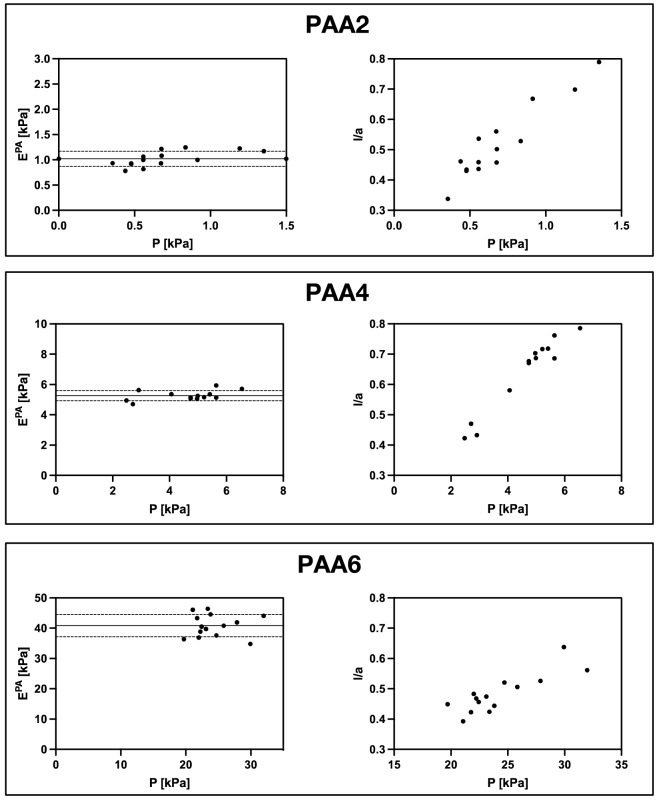
Elastic moduli of different PAA hydrogels measured by pipette aspiration test performed for different pressures. Data for three hydrogels are reported as example, relative to low, medium and high stiffness. Measurable *l/a* values are mostly > 0.3, to avoid large reading errors. The graphs indicate that calculated moduli are substantially constant with applied pressure. The lines in the figure on the left represent the mean value of E^PA^ and the standard deviation.

Given that in our experimental conditions a reliable reading of the aspiration length is achieved when *l/a* > 0.3, Eq. (7) was used to calculate E^PA^ values that are reported in Table 3. In particular, to verify that measures achieved in a range of *l/a* > 0.3 are substantially constant with applied pressures, the six PAA compositions were measured at different pressures. The results are shown in Fig. 6 (graphs on the right) in which each dot represents a single measure taken at a specific value of pressure. Then the elastic moduli E^PA^ are calculated for each value of *p* and *l/a* and reported in Fig. 6 (graphs on the left). Mean values and standard deviations are reported in Table 3, together with the minimum and maximum values of pressure used (and the respective ratio *l/a*).

**Table 3:** Minimum and maximum values of ratio *l/a* (and relative pressures) measured by micropipette aspiration for each gel. The mean values of the elastic moduli E^PA^ calculated for each value of *p* and *l/a* is reported together with their standard deviation.

	l/a min [µm]	l/a max [µm]	p min [kPa]	p max [kPa]	E^PA^ [kPa]	ΔE^PA^ [kPa]
PAA1	0.384	0.735	− 0.20	− 0.08	0.20	0.02
PAA2	0.338	0.789	− 1.35	− 0.35	1.02	0.15
PAA3	0.345	0.755	− 2.47	− 0.88	2.20	0.14
PAA4	0.423	0.786	− 6.55	− 2.48	5.27	0.33
PAA5	0.421	0.798	− 9.93	− 3.84	8.09	0.53
PAA6	0.393	0.638	− 31.98	− 19.71	40.85	3.69

### Comparison of methods to determine the elastic modulus

**Figure 7:**
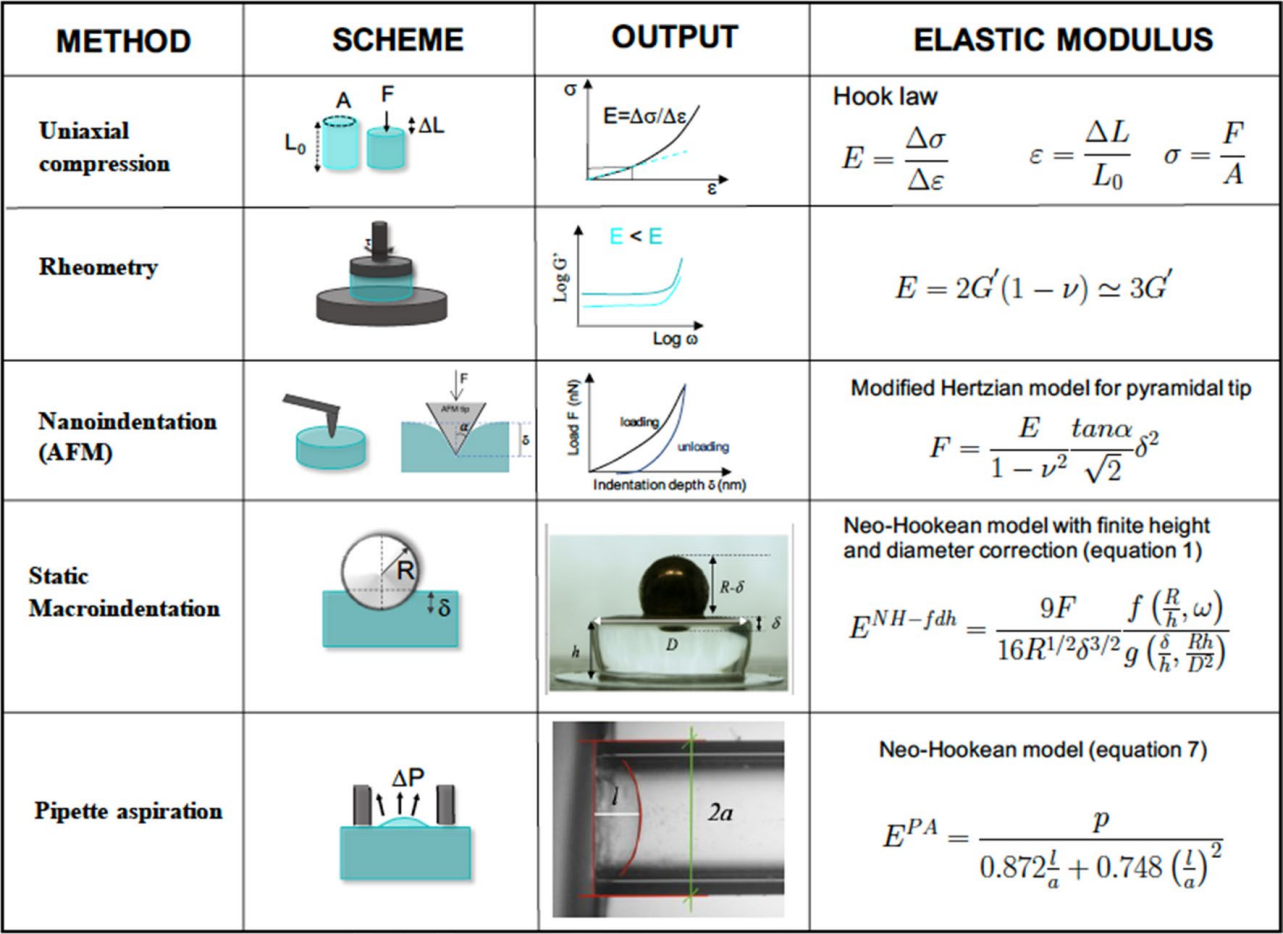
Schemes of the stresses applied to the hydrogels in the different techniques (column 2), experimental output achieved from the measurements (force/strain relationship or deformation state image, column 3), their relationship with the final result (column 4), the Young elastic modulus E.

**Figure 8:**
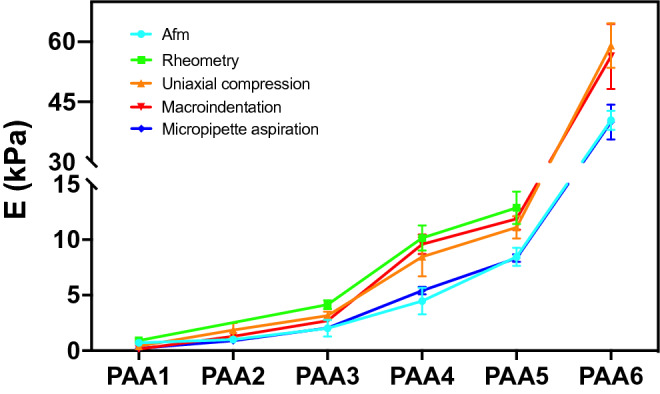
Elastic modulus of PAA hydrogels measured with micro indentation AFM (*sharp tip, from ref^3^), static macro sphere indentation, rheometry, pipette aspiration and uniaxial compression tests. Numerical data are reported in Table 4. In Fig. S8 errors data are visible.

To test the reliability of our methods, we compared the E values measured on the same six PAA hydrogels by static macroindentation and micropipette aspiration with those measured with traditional methods, adopting more expensive equipment and operating at lower throughput. First, the values we obtained with either method were nicely matching those expected by the six PAA formulations, that were indeed chosen from the recipes of Engler et al.^11^, who empirically derived such formulations using AFM measurements. We further experimentally supported the validity of our methods by comparing them with uniaxial compression (Fig. S4) and parallel plate rheometry (Fig. S7). Figure 7 provides a schematic representation of all the techniques involved in the comparison. A direct comparison between all these measurements is offered in Fig. 8 and Table 4. From the comparison, we found a consistent trend: the elastic modulus increases with the amount of crosslinking and a maximal 2/3-fold variation between elastic moduli calculated with the different techniques is observed over the same gradient of rigidities, except for the softest hydrogel.

**Table 4:** Comparison of PAA Hydrogel elastic modulus measured with different techniques. For static macroindentation data of E^NH-fhd^ are taken from Table 1. Data for micropipette aspiration EPA are calculated for a minimum of 10 measurements made on the same gel and ΔE represents the standard deviation.

Hydrogel Name	AFM from	Pipette aspiration $E^{PA}$	Macro-indentation $E^{NH - fdh}$	Rheometry	Compression
PAA1	0.71 ± 0.24	0.21 ± 0.02	0.14 ± .0.01	0.91 ± 0.02	0.40 ± 0.14
PAA2	1.16 ± 0.54	0.89 ± 0.11	1.3 ± 0.09		1.85 ± 0.64
PAA3	2.01 ± 0.75	2.04 ± 0.27	2.69 ± 0.24	4.14 ± 0.04	3.15 ± 0.35
PAA4	4.47 ± 1.19	5.41 ± 0.34	9.58 ± 0.88	10.14 ± 1.13	8.45 ± 1.77
PAA5	8.44 ± 0.82	8.33 ± 0.34	11.86 ± 0.97	12.87 ± 1.46	11.10 ± 0.99
PAA6	40.40 ± 2.39	39.94 ± 4.39	56.37 ± 8.15		59.05 ± 5.59

Of note, these setups have proved to be suitable to perform a large set of measurements rather quickly (up to 1 measurement in less than 5 min and 10 min for micropipette aspiration and static macro-indentation respectively, for the less challenging samples), while a minimum of 15–20 min is generally required for AFM and rehometry. The rapid measurement is important also to avoid drying of the hydrogel.

**Figure 9:**
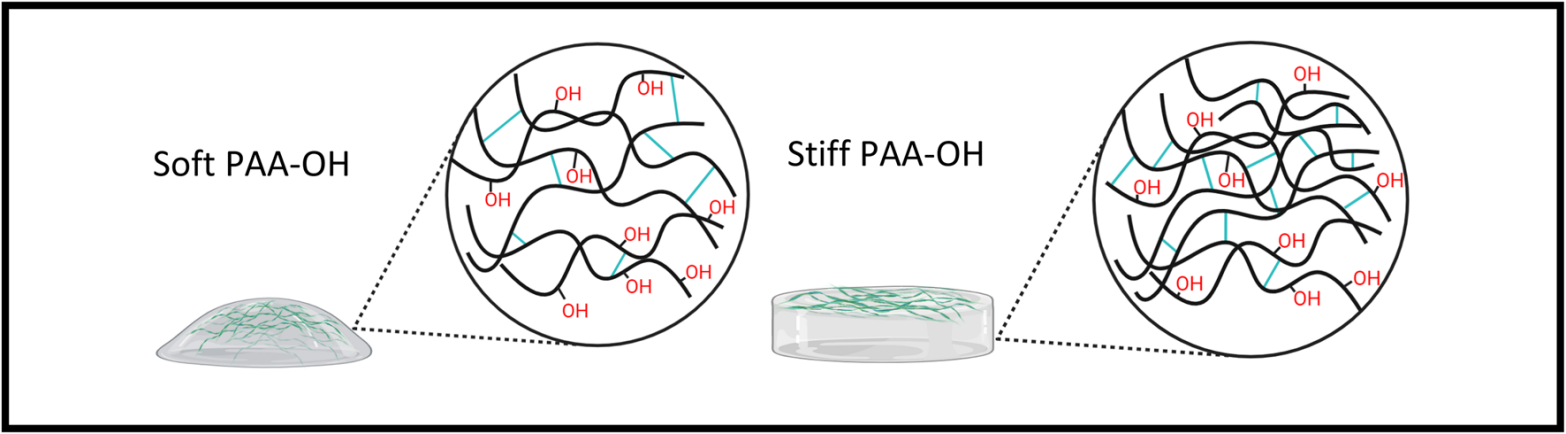
Scheme of PAA-OH hydrogels soft (left) and stiff (right).

**Figure 10:**
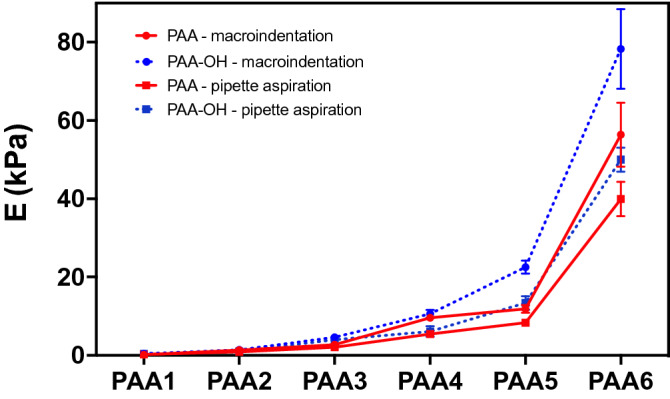
Elastic modulus of PAA and PAA-OH hydrogels measured with micropipette aspiration and macroindentation. Mean values and standard deviations are reported in Table S4.

As proof-of-concept of the applicability of our methods to a different material, we prepared an OH-functionalized PAA hydrogel, as previously reported^31^ (PAA-OH, Fig. 9, and Table 1). PAA-OH formulations are prepared replacing part of the AA of PAA formulations with a new monomer, N-hydroxyethyl acrylamide (HEA), copolymerized in different concentrations and molar ratio. The presence of highly polar group OH, as shown in Fig. 10 and Table 4, is sufficient to modify the mechanical properties of PAA-OH in respect to PAA, of twofold (e.g., from 2 to 4 kPa, that is within the range of biological significance), in spite of the fact crosslinking degree between the PAA and PAA-OH gels should be identical. This confirms the effectiveness and sensitivity of approach.

**Figure 11:**
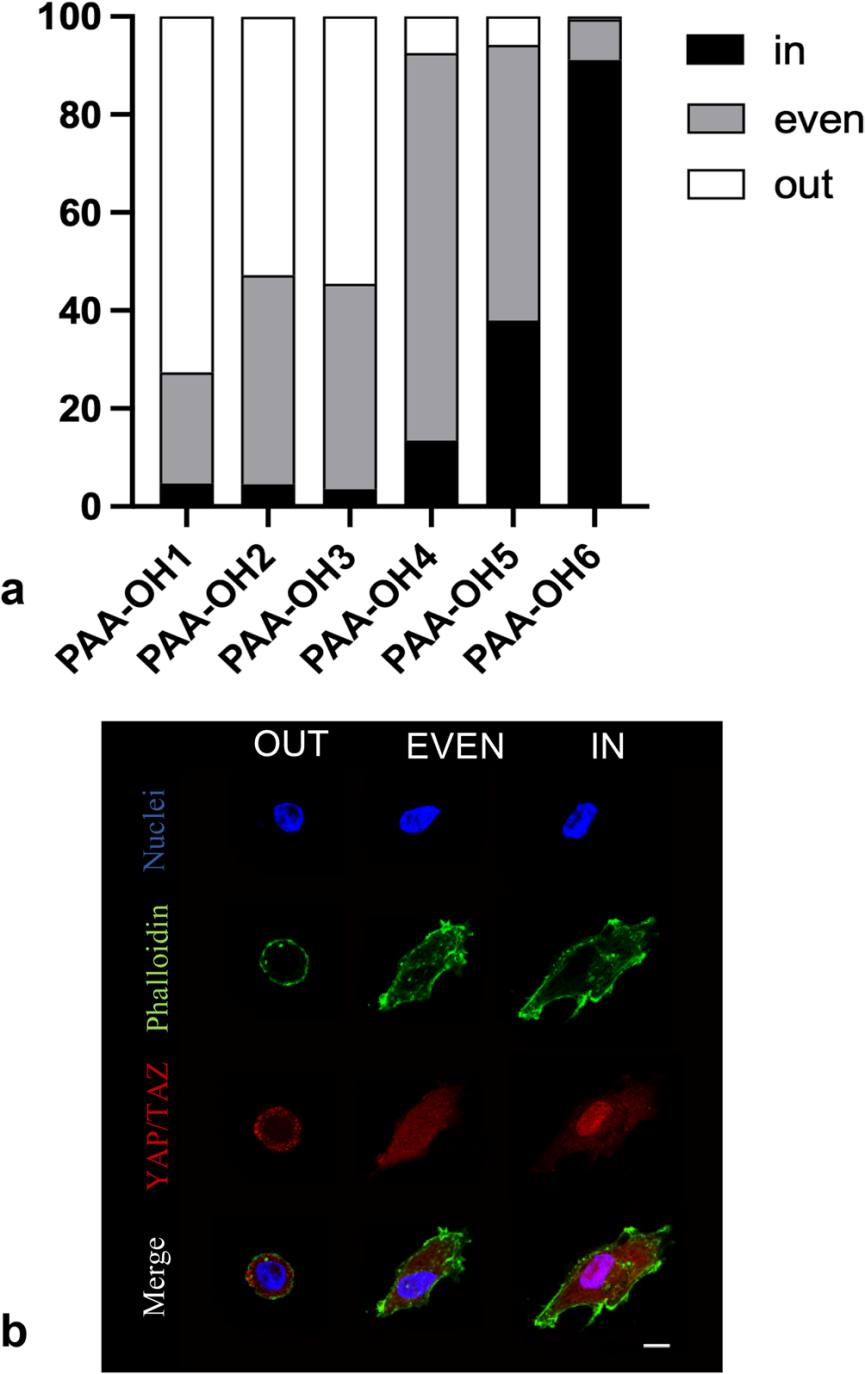
(**a**) Quantifications of the percentage of MFC10A cells displaying nuclear (in), cytoplasmic (out) or even YAP/TAZ subcellular localization, after seeding on the indicated substrates. YAP/TAZ immunofluorescence was performed on MCF10A cells seeded on PAA-OH hydrogels of five different stiffness. YAP/TAZ are nuclear and transcriptionally active in cells experiencing high-rigidities and progressively relocalize to the cytoplasm in cells experiencing more and more compliant substrates^2,32^. (**b**) IF stainings displaying the three different categories of YAP/TAZ localization employed for quantifications; out, YAP/YAZ staining (in red) is completely excluded from the area of nuclear staining (in blue), even, YAP/YAZ staining is evenly distributed throughout nucleus and cytoplasm, in, YAP/YAZ staining is exclusively co-localized with the nuclear counterstain. F-actin was stained with fluorescently-labeled phalloidin to serve as cell shape reference. Scale bar, 10 µm.

We next asked to if and to what extent measures obtained with macroindentation and micropipette aspiration really reflect biologically meaningful values of extracellular rigidity for mechanobiology applications. To address this issue, we measured YAP/TAZ nuclear vs. cytoplasmic localization in PAA-OH hydrogels for a gradient of PAA-OH rigidities. YAP/TAZ are transcription factors serving as universal readers of the cell mechanical state, and prime mediators of the mechanical challenges, turning them into gene-expression programs. YAP/TAZ are nuclear and transcriptionally active in cells experiencing high-rigidities and progressively relocalize to the cytoplasm in cells experiencing more and more compliant substrates^2,32^. Each PAA-OH gel tuned over a gradient of rigidity values was biofunctionalized with the same concentration of the adhesive ECM protein Fibronectin (FN) and used as substrate for adhesion of MCF10A cells. By immunofluorescence, shown in Fig. 11, we found that YAP/TAZ was robustly nuclear in hydrogels of measured E ≥ 13 kPa, to become gradually evenly distributed between the nucleus and the cytoplasm at intermediate stiffness values (around 6–13 kPa), and completely cytoplasmic or excluded in softest hydrogels (< 1 kPa). Of note, this gradient of YAP/TAZ nuclear vs. cytoplasmic localization nicely parallels hydrogels’ rigidity values, allowing to appreciate differences in biological behavior which might arise from gels with the same crosslinking degree but different chemistries.

## Discussion and conclusions

Here we show the assembly and applicability of two straightforward and high throughput techniques to measure hydrogel elastic moduli. Classic PAA hydrogels and modified PAA-OH gels have been used as playground for our studies. That said, we believe that the theoretical and practical tools here developed may serve well other hydrogel chemistries and formulations. Static macroindentation is particularly easy to set up and use: this method should be considered as a “bulk” mechanical test, giving that the indentation reaches a gel depth up to 1 mm. By FEM simulations we obtained Eq. (1) to calculate the Young’s elastic modulus from experimental data. Equation 1 has been developed starting with a modified Hertz equation^12^, that we have implemented for macroindentation to account for the finite diameter of the gel. This is particularly important if one needs to prepare samples of small dimensions, as it is often the case for specimens prepared for laboratory experiments. Thanks to Eq. (1) hydrogel stiffness can be calculated by performing very simple static indentation tests at the macro-scale and applying the modified equation to calculate the elastic modulus. As far as the accuracy of macroindentation is concerned, mean values of E calculated with this method, provided values with a statistical maximum error of about 10%, making this test reliable over a large range of stiffnesses (Table 1).

Setting-up the micropipette aspiration apparatus is more challenging with respect to static macroindentation, but opens to a variety of applications in living tissues that are not possible with indentation procedures. However, there are currently little protocols that describe in detail how to assemble a micropipette aspiration apparatus, and this is where we advance. The apparatus allowed to measure the hydrogels in a range of stiffness covering that of living tissues. The elastic moduli were achieved mediating values calculated from different data of pressures *P* and aspiration length *l*, using a model already reported in the literature (Eq. 7). The experimental data provided mean values of E with an error of about 10%, confirming that the set-up has a good precision for all the six stiffnesses analyzed (Table 3). We have also seen that this method can be applied to a broader range of stiffness and sample geometry with respect to static macroindentation; in particular, to the softest and irregularly shaped samples. In fact, these traits represent a source of inaccuracy for a macroindentation test due to added uncertainty in image analysis and diminished applicability of the theoretical equations, which are based on a smooth surface.

Validation of the two methods here described has been made by comparing E measurements obtained by means of three other commercial, more sophisticated and less cost-effective techniques. As shown in Fig. 8 we find a very consistent trend of data. That said, some differences could be appreciated between stiffness values measured with distinct methodologies, and various factors can be accounted for such variations. First, similarly to our macroindentation procedure, compression and rheometry predominantly measure bulk behavior^9^. Conversely, AFM, conceptually similar to microindentation, probe the specimen in a more superficial region (of the order of few μm)^24^. Micropipette aspiration also probes the gel surface although through a deeper depth (100–200 μm)^27^. To account for these differences, it should be considered that the gel surface is known to be different from the bulk^33,34^, typically due to weaker cross-linking of the polymeric superficial structure. Such difference explains why AFM and micropipette aspiration provide systematically smaller values of E. An additional cause of variation is that the elastic modulus, except for compression and rheometric test, is calculated through mathematical models that, although accurate, remain an approximation, and not a direct measure. Irrespectively of these considerations, the reassuring fact emerging from our analyses is that the values obtainable with our simple methods do not deviate significantly from those achievable by the other techniques, as such validating their applicability to test hydrogel rigidities. It is also worth noting that our methods enable to overcome some drawbacks sometimes intrinsically associated to excessive sensitivity of such more sophisticated methodologies; for example, these drawbacks include low throughput, the fact that surface impurities can modify AFM readings along with difficulties associated to maintain constant hydration during measurements^16,25^.

Finally, we have validated our measurements through YAP/TAZ nuclear localization, as such assuring that differences detected by biophysical measures do impact on living cells by imparting graded levels of mechanotransduction. In sum we believe this work should advance on the popularization of hydrogel fabrication for many applications in which physical cues are investigated at the cell biology level.

## Methods

### PAA and PAA-OH hydrogel synthesis

Acrylamide (A) solution (40% wt/V in water), bisacrylamide (BA) solution (2% wt/V in water) and water are mixed in the proper ratio to obtain the prepolymer solution. For PAA-OH part of the AA was substituted with HEA to a final molar concentration of 0.1. The solution is then degassed for 15 min at 0.1 bar, to remove the oxygen dissolved in the solution which would inhibit the polymerization. Ammonium persulfate (APS) dissolved in water (10% wt/V or 20% wt/V for PAA1-3 and PAA4-6 respectively). Once completely degassed, the monomer solutions are mixed with 1% V/V of the APS solution and 0.1% V/V of Tetramethylethylenediamine (TEMED). The final solution is then mixed and poured inside polystyrene multiwells (diameter 11 mm) cut to achieve a height of 4 mm or PDMS gasket (internally covered with a Kapton tape) for macroindentation or micropipette respectively. Both multiwell plate and PDMS molds were sealed with a glass coverslip functionalized with an adhesive silane. For cell seeding PDMS gaskets with an internal diameter of 20 mm and height of 250 μm are used. Functionalized coverslips are then used to seal the molds. Once polymerized (about 10–15 min), the gels are detached from the molds and placed in a petri dish submerged with milliQ water. Gels are left overnight to reach the swelling equilibrium before performing the mechanical test.

### Adhesive silanization

Glass coverslips with a diameter of 24 mm are washed with isopropanol and acetone and air dried to remove any residual grease or powder on the surface. After activation with a Bunsen flame, they are silanized 15 min with a solution of 3-(trimethoxysilyl)propyl methacrylate (TMSPM) composed by pure ethanol (950 μL), glacial acetic acid (50 μL), TMSPM (20 μL). The coverslips are then washed three times with pure acetone and air dried.

### PAA protein functionalization

Gels are placed in a multiwell dish inside the sterile hood and UV sterilized for 15 min. Once the sterilization is completed, a 1.5 mL of a fibronectin solution in 1X PBS (25 μg/mL) is pipetted inside each well. The gels are put at 37 °C inside an incubator overnight. The day after, the gels are extensively washed with sterile 1X PBS to remove any excess of fibronectin and residual monomers.

### Cell seeding

Cell line (immortalized mammary gland cells, MCF10A, a gift from F. Miller, Karmanos) are seeded pipetting 200 μl of cell suspension in culturing medium (concentration 200,000 cell/ml) on the surface of each hydrogel. When the adhesion of the cell to the substrate is completed, the wells can be flushed with culture medium submerging the whole hydrogel.

### Rheometric analysis

An amplitude sweep was preliminary preformed to assess the range in which the shear stress–strain response is linear thus identifying the adequate magnitude of the shear amplitude for the subsequent frequency sweep. Two circular cover glass (25 mm in diameter) where attached to both end of cylindrical hydrogel samples. Hydrogels were equilibrated in PBS for at least 3 days prior the rheological test. Samples were placed between the plates of an Anton Paar MC302 rheometer and the glass slides were attached to the rheometer plates (upper plate 25 mm diameter) with a double-sided adhesive tape. The amplitude sweep was performed in the 0.01–1% shear strain at a frequency of 1 Hz. Hydrogels displayed a constant storage and loss modulus in the tested stain range. Frequency sweep, in the 0.01–10 Hz interval at 0.1% constant strain, was then performed on the hydrogel samples. Seven experimental points were acquired per each frequency decade. Tests were performed at 37 °C in wet conditions.

### Uniaxial compression

Hydrogel samples were prepared as per the rheometric analyses. Samples were secured between the plates (upper plate 25 mm diameter) of an Anton Paar MC302 rheometer. Upper plate was lowered in a step wise manner until a total compression of 20–25% was reached. Approximately, 14 data points were acquired per each compression test. A time lag of 30 s was applied before acquiring the normal force in order to stabilize the output of the load cell. Tests were performed at 37 °C in wet conditions.

### Static macroindentation apparatus set-up

A light box with a led light on the rear (schematized in Fig. S1 e.g. Whitebox plug and play lightbox: https://lightbox.sg/) an Olympus (E510) reflex camera with a focal length of 42 mm and a set of spheres made of different materials (Table S1) are used. Since the stiffer gels were not indented enough by the heaviest sphere, the tests on PAA5 and PAA6 were performed with an indenter of 7585 mg prepared by attaching the glass sphere on a plastic plate sliding on vertical guides (Fig. S1).

To perform the mechanical test, the gel is placed on a stage inside the light box and the excess of water on the surface is absorbed with blotting paper. The selected sphere is then gently placed on the center of the free surface of the gel. It is important to avert any downward push while placing the sphere on the gel to avoid any external contribution to the indentation. To correctly perform the mechanical measurement, it’s very important to properly chose the dimension and weight of the indentation sphere used as described in Table S2.

**Figure 12:**
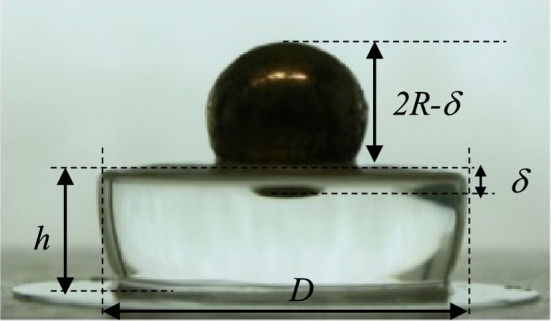
Example of image analysis for the determination of parameters *δ, h, D* with a MATLAB program.

The images taken during the analysis are analyzed using a custom MATLAB code that guides the user to the identification of the parameters required to perform the analysis. The code has been written to analyze the height of the gel, the diameter of the sphere and the dimension of the non-indenting part of the sphere as shown in Fig. 12. Keeping the diameter of the sphere as a reference, the code provides the indentation depth. The data acquired from the image are then used to calculate the Young modulus of the hydrogel using the model optimized by the FEM analysis.

The obtained value represents a single measure, that is generally repeated at least 10 times to improve the reliability of the final estimation of the elastic modulus E^NH-fdh^. Mean values of the elastic moduli and relative standard deviation are reported in Table 4 and Fig. 8 (for PAA) and Table S4 and Fig. 10 (for PAA vs PAA-OH). In the figures the error bars represent the standard deviation. We have also verified that sets of measures made on two or three different gels (with the same composition) have a variation within the errors calculated in each set of measures.

### Micropipette aspiration apparatus set-up

#### Structure design

The structure of the micropipette testing instrument is divided in two main parts: a box for the electronic circuits and sensors (3 in Fig. S5) and the positioning section (4 in Fig. S5) which includes the sample and pipette holder, for the mounting of the micropipette setup on any inverted microscope.

The circuits enclosure design is based on a previously published open source design (https://www.thingiverse.com/thing:2807485) properly modified to suits the project requirements while the positioning system has been designed ex novo.

The design of this section has been done to fit the sample and the pipette into a 100 mm petri dish in order to keep alle the required parts submerged in the liquid medium.

All the 3D files have been designed using a CAM/CAD software (Autodesk Fusion 360) and then printed with a desktop FDM 3D printer (Creality Ender 5). All custom 3D models, used to setup the apparatus, are available at the following link:

https://drive.google.com/drive/folders/1MhXHN8KKk4UGJXTNko7Sve775J6lc0KZ?usp=sharing.

**Figure 13:**
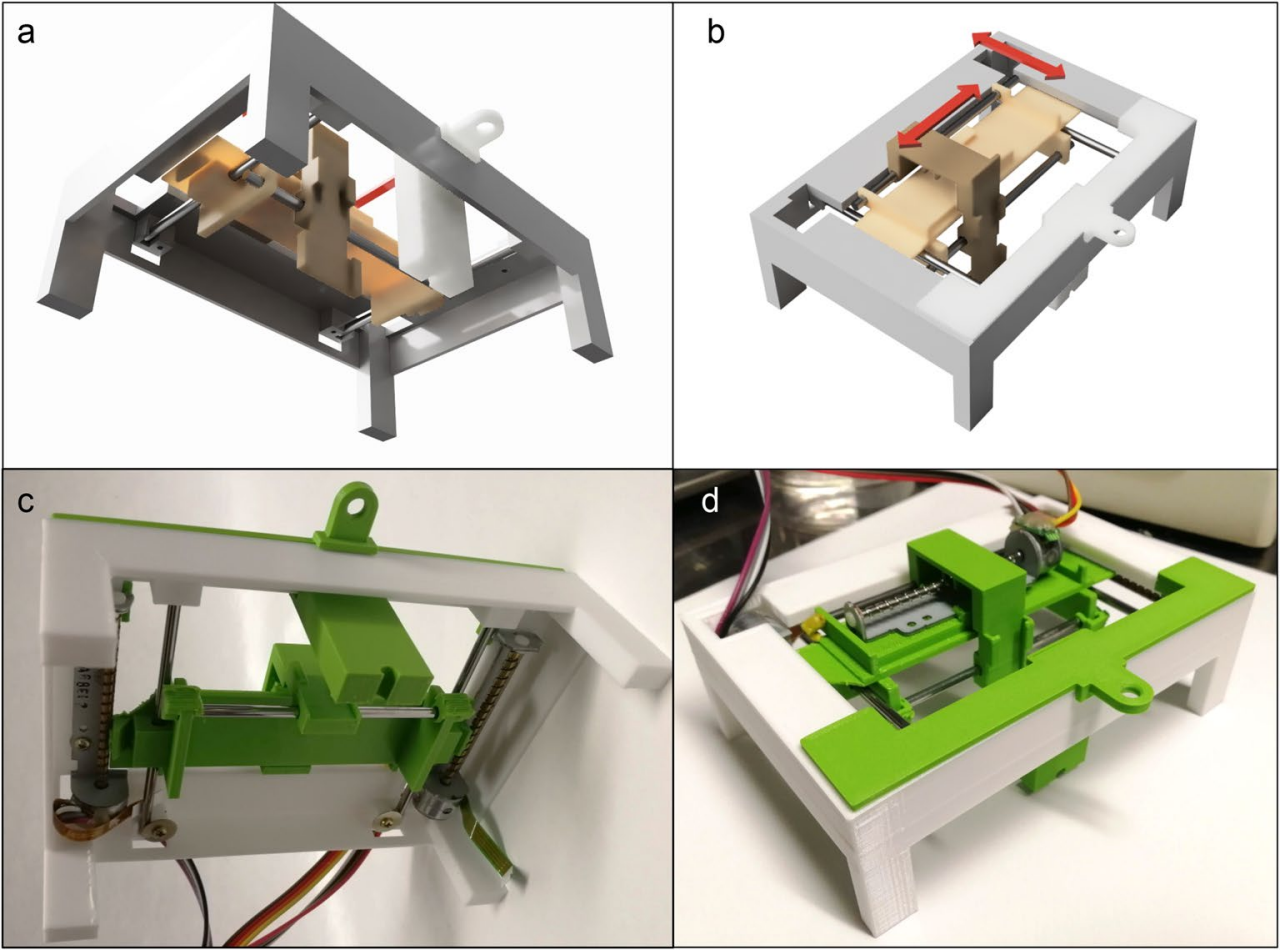
(**a**, **b**) Renders of the 3D printed files. (**c**, **d**): pictures of the micropipette positioning system and micropipette holder.

The testing system consisting of the holders and the micromanipulator is shown in the Fig. S5 and, in more detail, in Fig. 13.

### Electronic circuit and control

**Figure 14:**
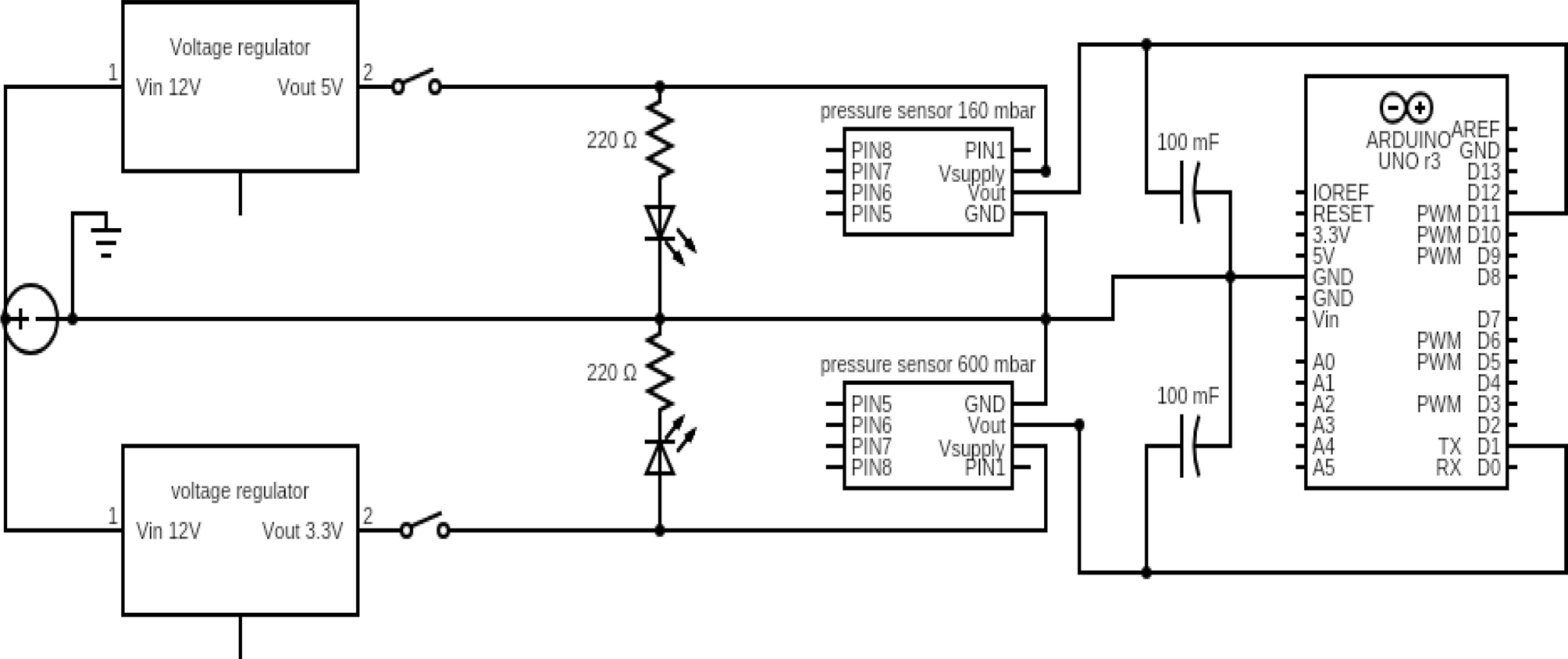
Scheme of the electronic circuit for the pressure sensors control.

The electronic control over the micropipette pipette was obtained using two open-source microcontroller boards (Arduino UNO). One of the boards is used to record the pressure values. To do this, an electronic circuit has been created as reported in Fig. 14. A DC-DC converter is used to supply a stable voltage of 5 V or 3.3 V to the sensors allowing them to minimize the current variations. Two 0.1 μF capacitors are used between the sensors output and the ground to filter the analog signals, thus decreasing the voltage variations in the output.

The sensors chosen for the system assembly are two analog differential pressure sensors with two different range sensitivity produced by Honeywell (Honeywell HSCDRRN600MDAA3 for pressures from − 600 mbar to 600 mbar and Honeywell HSCDRRN160MDAA5 for pressures between − 160 mbar and 160). The achievable precision of the pressure measurements is limited by the resolution of the analog to digital converter (ADC) of the microcontroller.

The second Arduino board is used to provide the spatial positioning of the sample. This is done controlling three linear stepper motors (SM15DD) through a commercially available extension board (CNC shield v3.0) that can control up to four motors using 4 stepper drivers (for example Polulu A4988 were used in our apparatus).

### Mechanical characterization

The sample is mounted on the sample holder with the free surface perpendicular to the glass capillary.

To improve the analysis of the image, a dye (water insoluble) was poured on the surface of the softer samples.

Then, the positioning system is placed on the stage of an inverted microscope inside a plastic petri dish which is subsequently filled with the liquid medium required for the analysis. It’s important to completely submerge the sample to avoid any optical diffraction.

For the analysis, the sample is moved towards the glass capillary until a full contact between the surfaces is achieved and the initial pressure is recorded. An increasing negative pressure is applied to the sample through the syringe pump until a small convexity is observed through the microscope. The syringe pump is stopped, and the final pressure is recorded while a picture of the aspirated length is taken with the microscope software.

**Figure 15:**
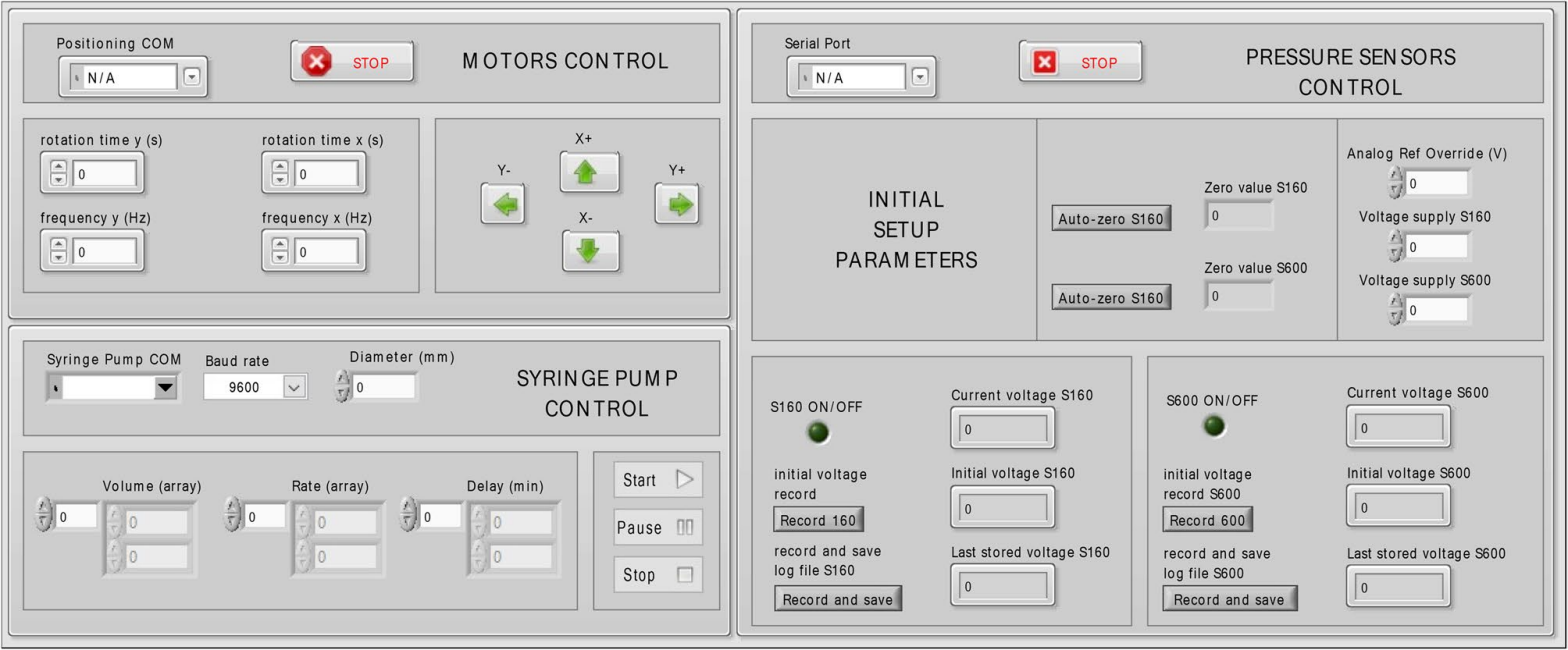
LABVIEW interface to control the micropipette apparatus.

To control the positioning system, the sensor output and the syringe pump a LABVIEW program is used, with a user graphic interface designed to have the complete control over all the steps of the analysis (Fig. 15). The code comprises the recording of the initial pressure measured at t = 0 and of the final pressure obtained over the averaging of 10 measurements performed every 50 ms at a fixed value of pressure. Once the measurement is concluded the software automatically saves a log file with all the required parameters for the subsequent analysis. The obtained value represents a single measure, that is generally repeated at least 10 times to improve the reliability of the final estimation of E^PA^: the pressure used for each measure is not necessarily the same. The corresponding values of elastic modulus are then calculated. Mean values and relative standard deviation are reported in Fig. 8 and Table 4 (for PAA) and in Fig. 10 and Table S4 (for PAA vs PAA-OH). The values and error bars in the figures represent the standard deviation. As in the case of macroindentation, here we found that sets of measures made on two or three different gels (with the same composition) display a variation within the errors calculated in each set of measures, indicating that gel-to-gel variation is negligible if compared with the errors of the mechanical measurements.

**Figure 16:**
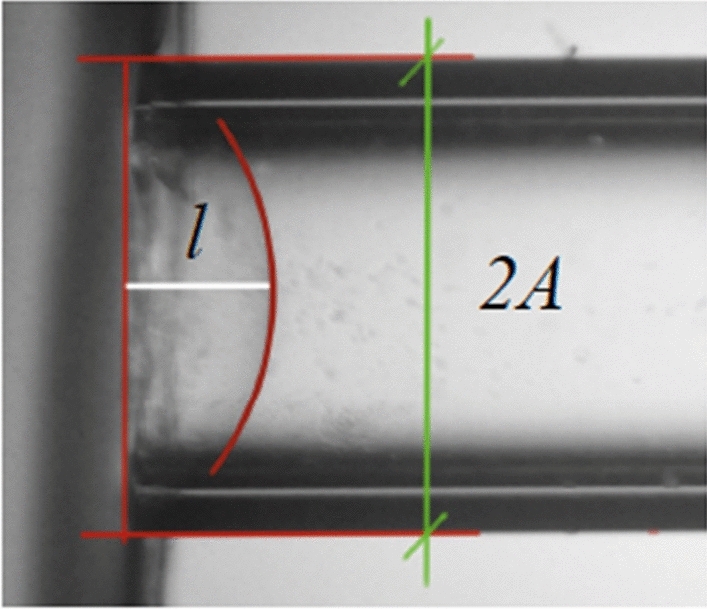
Example of the image analysis. l is the aspiration length and 2A is the external diameter (1 mm) of the glass pipette which is used as a reference by the MATLAB code.

By image analysis, the length of the aspirated portion of the sample is measured by a MATLAB program that guides the user for the quantification of the maximum displacement at the center of the pipette cross section (Fig. 16, white line).

### Immunofluorescence

Immunofluorescence was performed on PFA-fixed cells. Primary antibody was YAP/TAZ (Santa Cruz Biotechnology no. sc-101199). F-actin was stained with Alexa Fluor 488 Phalloidin (Thermo Fisher Scientific). Secondary antibody (1:200) were from Molecular Probes. Samples were counterstained with ProLong-DAPI (Molecular Probes, Life Technologies) to label cell nuclei. Confocal images were obtained with a Leica TCS SP5 equipped with a CCD camera and analysed using Volocity software (PerkinElmer, version 5.5.1).

### FEM simulation

The response of the neo-Hookean solid is governed by the strain energy function:

$$\phi = C_{10} \left( {\overline{I}_{1} - 3} \right) + \frac{1}{D}\left( {J - 1} \right)^{2}$$

where, Φ is the strain energy density per unit volume of the undeformed configuration. *C*_10_ and *D* are the material parameters, *J* is the deformation volume ratio. The initial bulk modulus *K*_o_ is related to D as K_o_ = 2/D and the initial shear modulus *µ*_o_ is related to *C*_10_ as *µ*_o_ = 2*C*_10_. The analysis is performed in two-dimensions, since the gel specimens have cylindrical shape and can be treated as axisymmetric. The gels are thus discretized with 6-node axisymmetric second-order triangular CAX6M-type elements. The hydrogel is meshed with finer elements closer to the center of the indent and is gradually meshed coarser along the depth and width of the hydrogel. The minimum and maximum element sizes are 0.001* h* and 0.1* h* respectively. The total number of elements used for the simulations is 30,000. A mesh sensitivity analysis was performed, but not presented here for brevity. In the simulations, the outer surface of the indenter and the top surface of the substrate are modeled as a contact pair. In order to prevent interpenetration between the two surfaces, the hard-wall interaction is implemented in the normal direction. The contact is assumed to be frictionless.

The equilibrium solution for the contact problem is obtained as follows: the total indentation is applied in several adaptive incremental steps. For each of the tentative incremental step, equilibrium is assumed to be reached when the force residual at all the nodes is less than 0.5% of the average nodal forces in the solid. If the solution has not converged within 16 iterations or if the solution appears to diverge, the tentative increment is reduced by a factor 0.25. If, instead, less than five iterations are required to reach convergence for two increments in a row, the subsequent incremental step is increased by a factor 0.5. In Fig. 4, the finite element mesh is shown along with the normal displacement field of the deformed hydrogel.

## Supplementary Information

Supplementary Information.
