# Supplementary material for: Simple yet effective methods to probe hydrogel stiffness for mechanobiology
Source: Sci Rep. 2021 Nov 22;11:22668. doi: 10.1038/s41598-021-01036-5 (PMC8608946; doi:10.1038/s41598-021-01036-5)
Supplement: Supplementary file 1 — Supplementary Information. [file 41598_2021_1036_MOESM1_ESM.docx]

**SUPPLEMENTARY MATERIALS**

**Figure S1: a)** scheme of the main parts of the microindentation apparatus and **b)** scheme of the overweight, used for stiffest gels, added to the sphere and running on two metal tracks (that perfectly fit the overweight holes) to avoid tilting of the overweight

**Figure S2:** images of the PAA gels indented with the selected spheres (see Table S2). For stiff hydrogels (PAA5 and PAA6) an overweight has been added to the sphere (Figure S1b).

**TABLE S1:** physical properties (weight and diameter) of the spherical indenter used

| **Indenter name** | **Indenter type** | **Weight (mg)** | **Diameter (mm)** | **Force (N)** |
| --- | --- | --- | --- | --- |
| **SP** | **Plastic sphere** | 46.7 | 4 | 0.46 |
| **SG** | **Glass sphere** | 165 | 4.9 | 1.62 |
| **SS** | **Steel sphere** | 700 | 5.5 | 6.86 |
| **SI** | **Glass sphere with an overweight** | 7580 | 4.9 | 74.4 |

**TABLE S2:** maximum and minimum values of *δ/h* ratio measured for each PAA gel (from a set of 10 measures) with different indenters (the physical properties of the indenters are reported in **Table S1**). In the table the mean value of *δ/h* ratio, the maximum and percentage error are reported. The indenter and relative *δ/h* ratio values used for each gel are selected in order to minimize the reading % error of the indentation ratio (last column of the table) and are evidenced in bold.

|  | **Indenter** | **δ/h** | **(δmax-δmin)/2** | **δ/h mean** | **Error %** |
| --- | --- | --- | --- | --- | --- |
| **PAA1** | **SP** | **0.195 - 0.227** | **0.016** | **0.211** | **7.6%** |
|  | SG | 0.32 - 0.38 | 0.03 | 0.35 | 8.6% |
| **PAA2** | SP | 0.059 - 0.089 | 0.015 | 0.074 | 20.3% |
|  | SG | 0.085 - 0.157 | 0.036 | 0.121 | 29.8% |
|  | **SS** | **0.24 - 0.274** | **0.017** | **0.257** | **6.6%** |
| **PAA3** | SP | 0.035 - 0.051 | 0.008 | 0.043 | 18.6% |
|  | SG | 0.084 - 0.11 | 0.013 | 0.097 | 13.4% |
|  | **SS** | **0.164 - 0.194** | **0.015** | **0.179** | **8.4%** |
| **PAA4** | SS | 0.073 - 0.089 | 0.008 | 0.081 | 9.9% |
|  | **SI** | **0.337 - 0.397** | **0.03** | **0.367** | **8.2%** |
| **PAA5** | SS | 0.064 - 0.088 | 0.012 | 0.076 | 15.8% |
|  | **SI** | **0.314 - 0.364** | **0.025** | **0.339** | **7.4%** |
| **PAA6** | SS | 0.038 - 0.062 | 0.012 | 0.05 | 24.0% |
|  | **SI** | **0.115 - 0.15** | **0.0175** | **0.1325** | **13.2%** |

**Figure S3:** The maximum depth of indentation for a given radius of curvature compatible with the assumptions of the Hertz theory. For specimens with D/h ratios of 1, 2 and 5, the curves give the limiting indentation depth normalized on the diameter above which classical Hertzian theory does not hold.

**TABLE S3:** Sample and hydrogel parameters used for the FEM simulation of macroindentation experiments.

| **Sample** | ***R* (mm)** | ***δ* (mm)** | ***h* (mm)** | ***F* (mN)** | ***D* (mm)** | **δ/h** |
| --- | --- | --- | --- | --- | --- | --- |
| **PAA1** | 2.0 | 1.0 | 4.5 | 0.46 | 13.7 | 0.23 |
| **PAA2** | 2.7 | 1.2 | 4.3 | 6.9 | 12.5 | 0.27 |
| **PAA4** | 2.7 | 0.8 | 4.0 | 6.9 | 11.5 | 0.19 |
| **PAA5** | 2.7 | 0.3 | 3.9 | 6.9 | 10.9 | 0.09 |
| **PAA6** | 2.4 | 1.3 | 3.6 | 74.4 | 10.6 | 0.36 |
| **PAA7** | 2.4 | 0.5 | 3.6 | 74.4 | 9.8 | 0.15 |
|  |  |  |  |  |  |  |


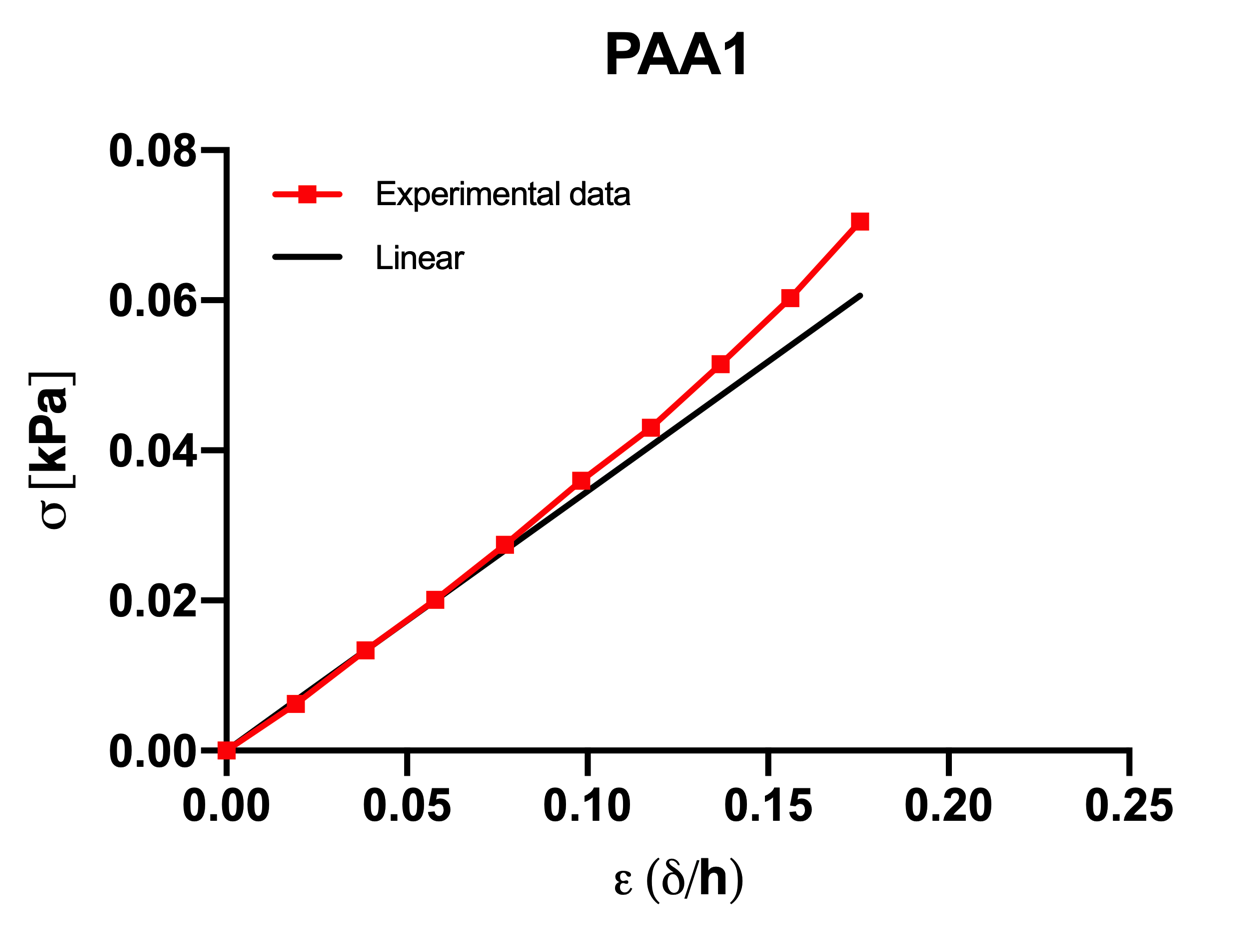

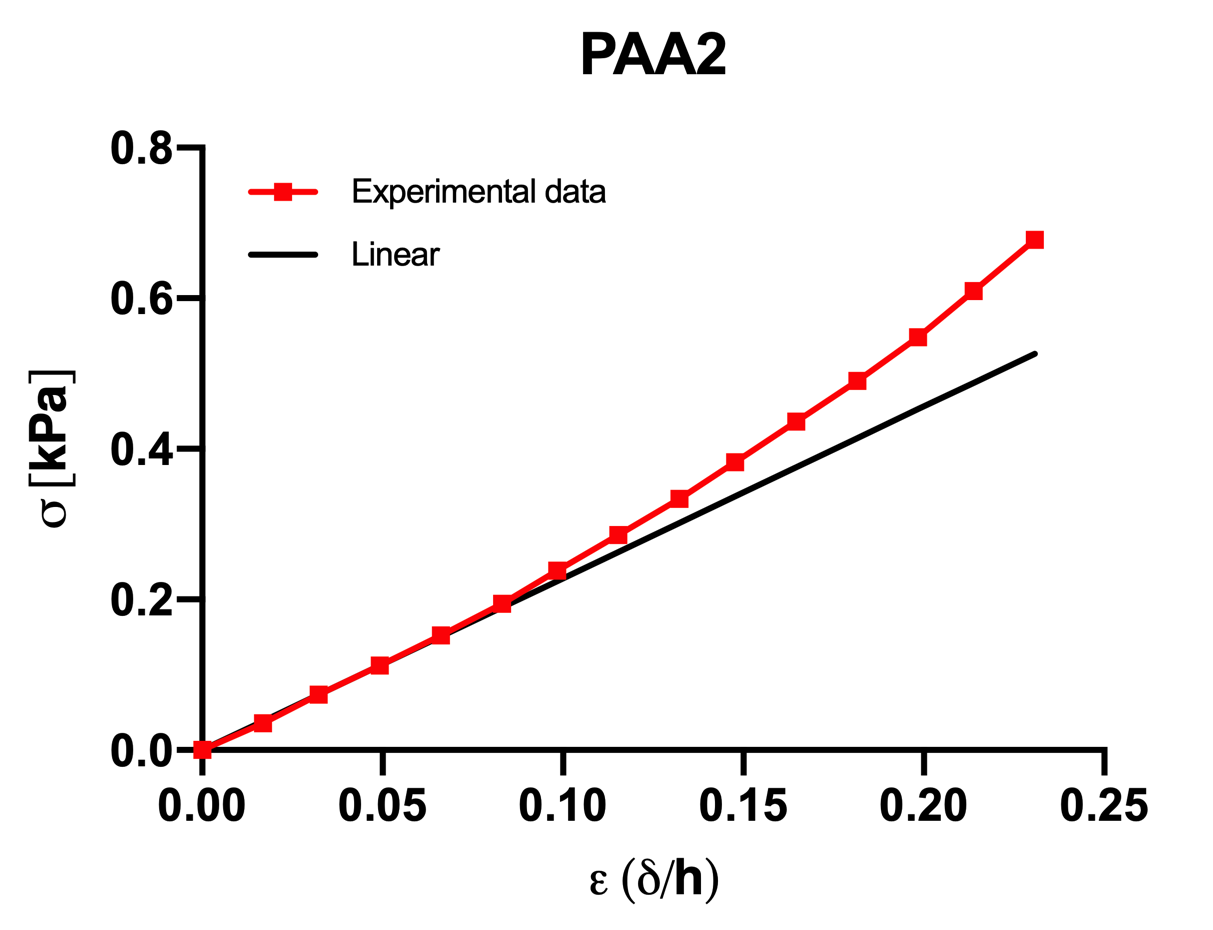

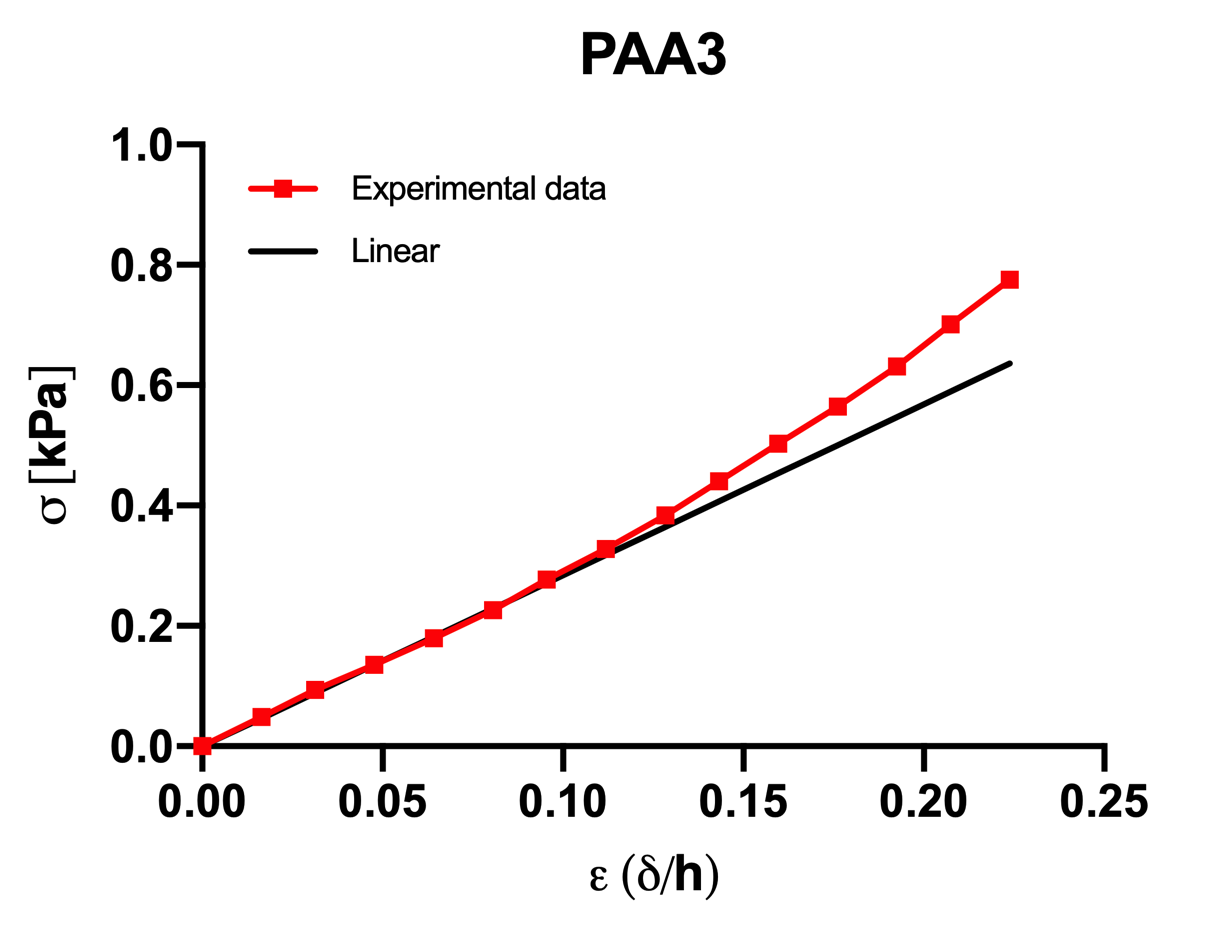

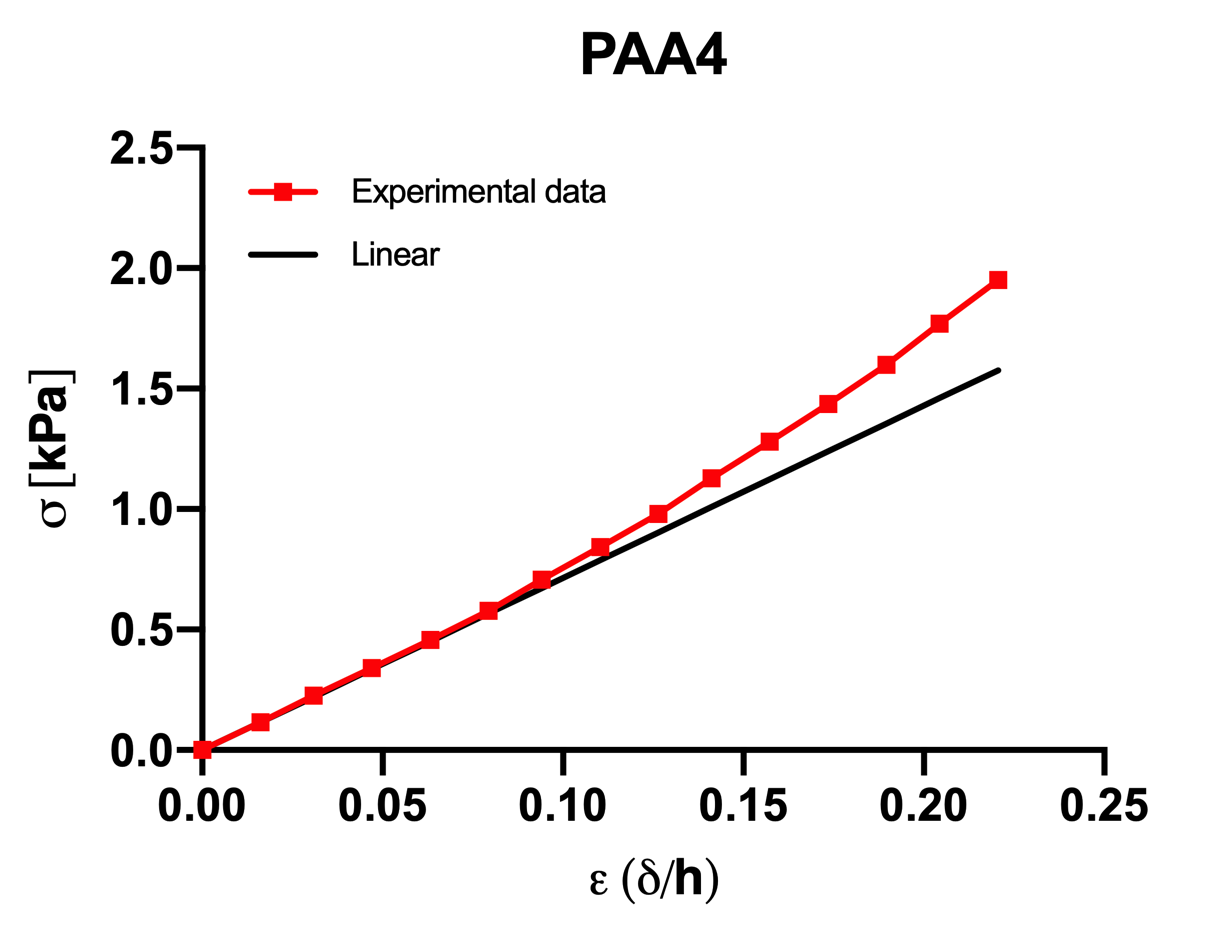

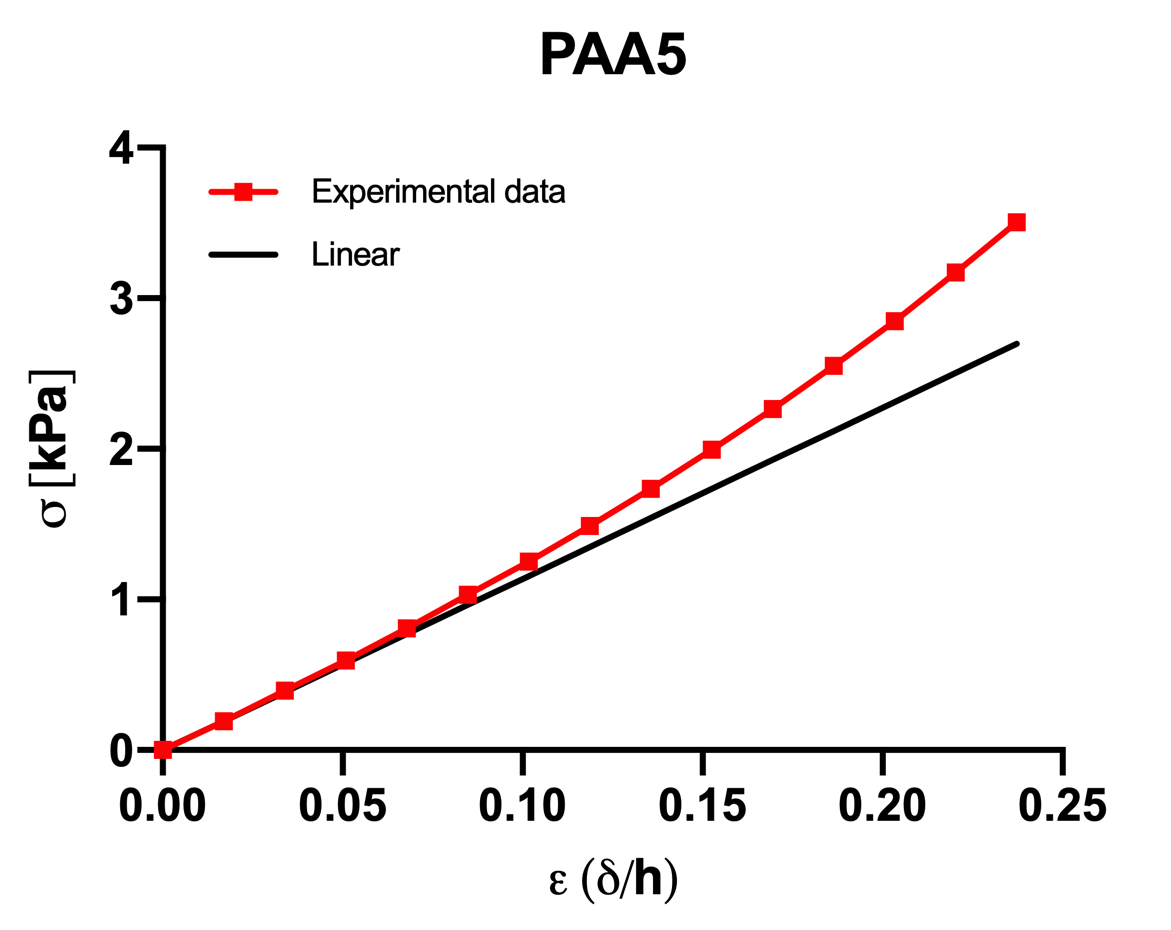

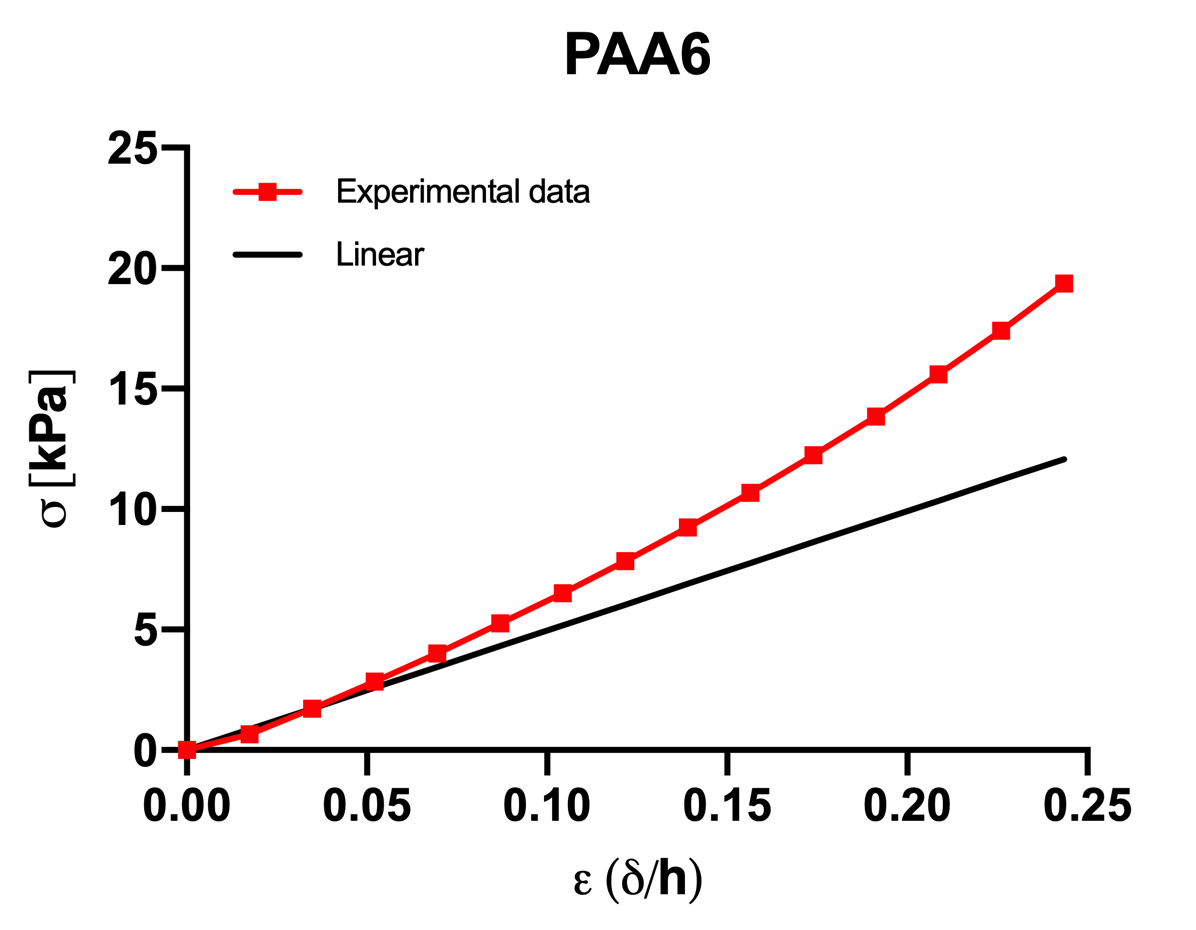


**Figure S4:** Example of uniaxial compression tests performed on the 6 PAA. The non-linear behavior of PAA is observed for deformation ε often <5%. In gels used for the macroindentation test, generally between 4 and 5 mm in height, any indentation that deform the surface of more than 500μm, and then display a ratio *δ/h* >0.01 are considered exceeding the 10% of deformation.

d

c

**
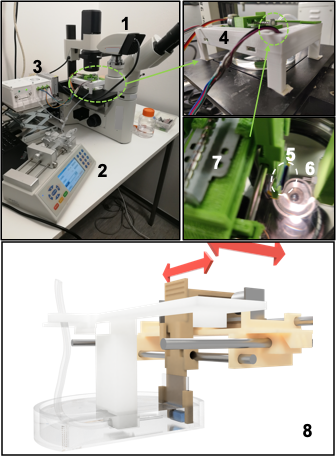
**

**Figure S5** Micropipette aspiration set-up overview. **1**: microscope **2**: syringe pump **3**: digital controller (of movement and sensors) **4**: sample, xy positioning system and micropipette holder **5**: sample **6**: micropipette **7**: positioning system **8:** rendering of the internal part of the system

**
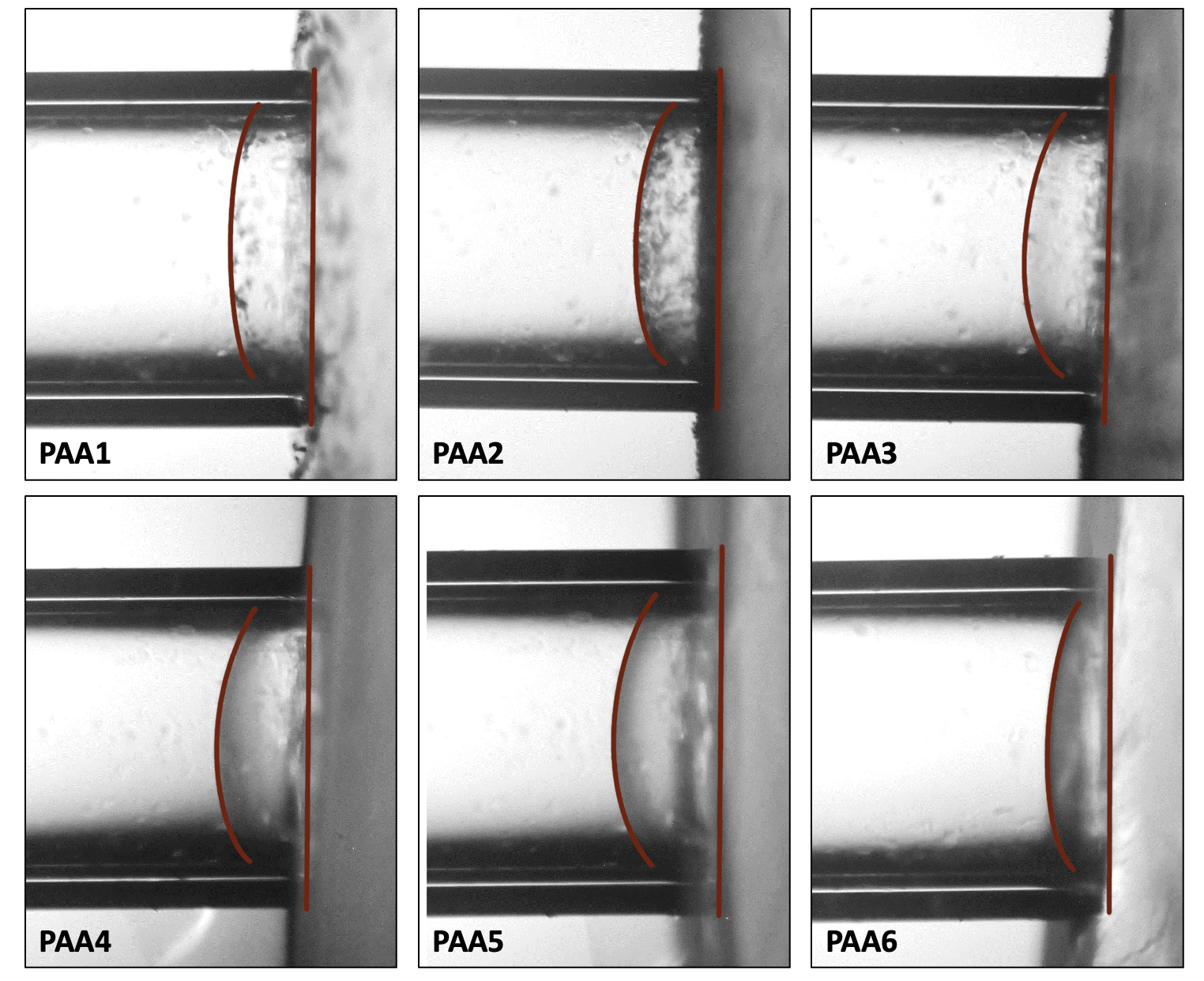
**

**S6:** Example of images of the 6 PAA gels during pipette aspiration. These images are used for further image analysis.

**Figure S7:** Rehometric measurements of PAA hydrogels, storage modulus (E’, left) and loss modulus (E”, right).


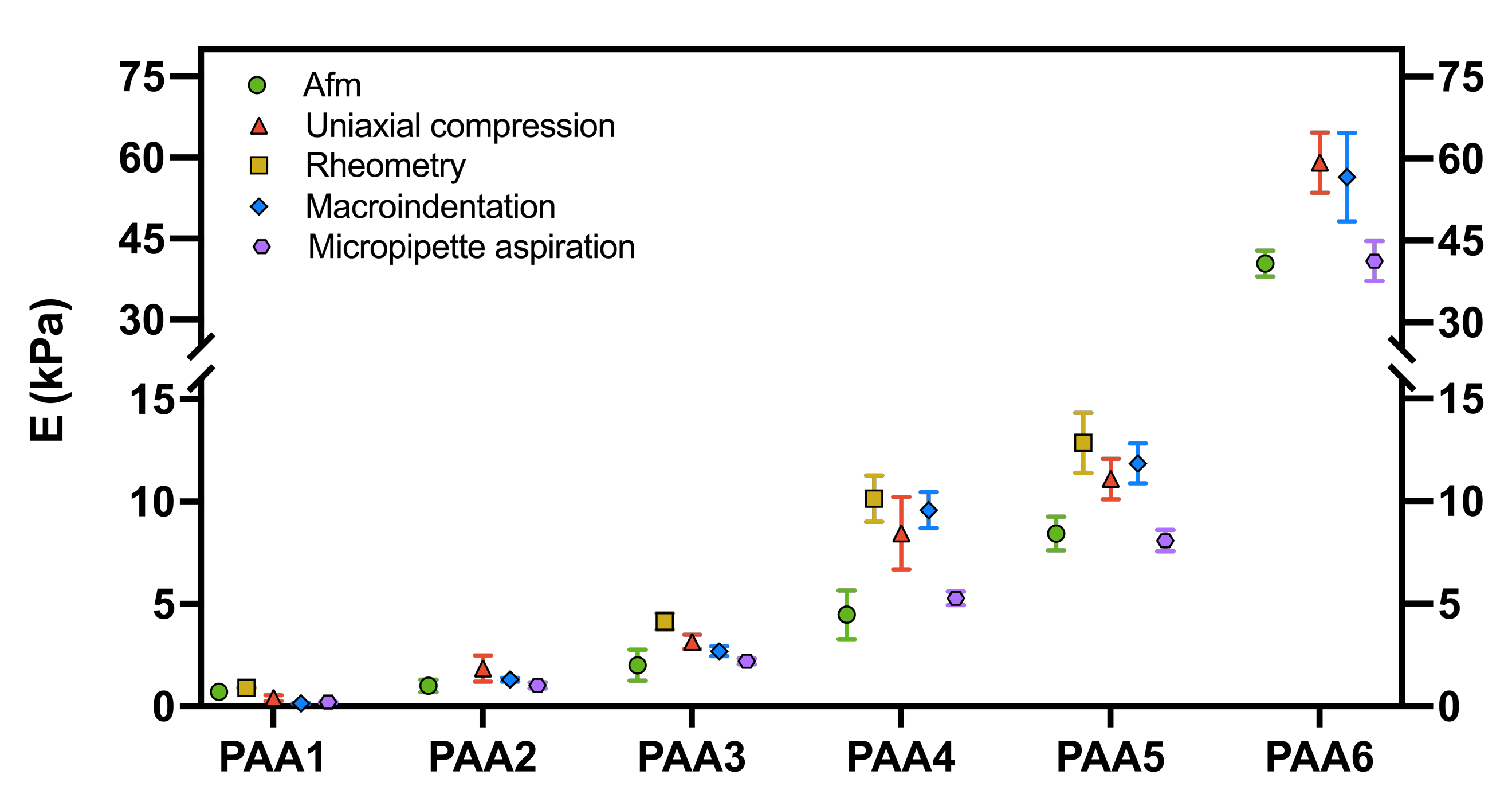


**Figure S8:** Elastic modulus of PAA hydrogels measured with micro indentation AFM (*sharp tip, from ref ^3^), static macro sphere indentation, rheometry, pipette aspiration and uniaxial compression tests. Numerical data are reported in **Table IV**

**TABLE S4:** PAA and PAA-OH Hydrogel elastic modulus measured with static macroindentation and micropipette aspiration. For PAA data are from **Table IV**. The procedure to calculate errors on E^NH-fhd^ and E^PA^ for PAA-OH are the same described for PAA (see method section).

| **Hydrogel Name** | **Pipette aspiration** $\boldsymbol{E}^{\boldsymbol{PA}}$ | **Macro-indentation** $\boldsymbol{E}^{\boldsymbol{NH}-\boldsymbol{fdh}}$ | **Hydrogel Name** | **Pipette aspiration** $\boldsymbol{E}^{\boldsymbol{PA}}$ | **Macro-indentation** $\boldsymbol{E}^{\boldsymbol{NH}-\boldsymbol{fdh}}$ |
| --- | --- | --- | --- | --- | --- |
| PAA1 | 0.21±0.02 | 0.14±.0,01 ^1^ | PAA-OH1 | 0.33±0.04 | 0.42±0.03 |
| PAA2 | 0.89±0.11 | 1.3±0.09 ^2^ | PAA-OH2 | 0.96±0.23 | 1.38±0.07 |
| PAA3 | 2.04±0.27 | 2.69±0.24 ^2^ | PAA-OH3 | 3.99±0.59 | 4.60±0.45 |
| PAA4 | 5.41±0.34 | 9.58±0.88^2^ | PAA-OH4 | 6.09±1.35 | 10.60±1.04 |
| PAA5 | 8.33±0.34 | 11.86±0.97 ^3^ | PAA-OH5 | 13.39±1.73 | 22.50±1.70 |
| PAA6 | 39.94±4.39 | 56.37±8.15 ^3^ | PAA-OH6 | 50.01±3.07 | 78.30±10.20 |
